# Unraveling Hidden Major Factors by Breaking Heterogeneity into Homogeneous Parts within Many-System Problems

**DOI:** 10.3390/e24020170

**Published:** 2022-01-24

**Authors:** Elizabeth P. Chou, Ting-Li Chen, Hsieh Fushing

**Affiliations:** 1 Department of Statistics, National Chengchi University, Taipei 11605, Taiwan; eptchou@g.nccu.edu.tw; 2 Institute of Statistical Science, Academia Sinica, Taipei 11529, Taiwan; tlchen@stat.sinica.edu.tw; 3 Department of Statistics, University of California at Davis, Davis, CA 95616, USA

**Keywords:** CEDA, Magnus effect, conditional entropy, heterogeneity, mutual information, Rosenberg Self-Esteem Scale

## Abstract

For a large ensemble of complex systems, a Many-System Problem (MSP) studies how heterogeneity constrains and hides structural mechanisms, and how to uncover and reveal hidden major factors from homogeneous parts. All member systems in an MSP share common governing principles of dynamics, but differ in idiosyncratic characteristics. A typical dynamic is found underlying response features with respect to covariate features of quantitative or qualitative data types. Neither all-system-as-one-whole nor individual system-specific functional structures are assumed in such response-vs-covariate (Re–Co) dynamics. We developed a computational protocol for identifying various collections of major factors of various orders underlying Re–Co dynamics. We first demonstrate the immanent effects of heterogeneity among member systems, which constrain compositions of major factors and even hide essential ones. Secondly, we show that fuller collections of major factors are discovered by breaking heterogeneity into many homogeneous parts. This process further realizes Anderson’s “More is Different” phenomenon. We employ the categorical nature of all features and develop a Categorical Exploratory Data Analysis (CEDA)-based major factor selection protocol. Information theoretical measurements—conditional mutual information and entropy—are heavily used in two selection criteria: C1—confirmable and C2—irreplaceable. All conditional entropies are evaluated through contingency tables with algorithmically computed reliability against the finite sample phenomenon. We study one artificially designed MSP and then two real collectives of Major League Baseball (MLB) pitching dynamics with 62 slider pitchers and 199 fastball pitchers, respectively. Finally, our MSP data analyzing techniques are applied to resolve a scientific issue related to the Rosenberg Self-Esteem Scale.

## 1. Introduction

A Many-System Problem (MSP) is a scientific study involving many complex systems [1,2,3]. Such systems are basically governed by the same dynamic principles with many complex tuning parameters that embrace hardly measured idiosyncratic characteristics. This nature of MSPs can be seen in almost all scientific areas and real-world research involving any sort of heterogeneity, such as study subjects of different ages and genders in psychology and neuroscience [4], many distinct households in microeconomics [5], and the well-known many-body problem in physics [6], just to name a few. It is notable that the name MSP is inspired by the many-body problem.

Data observed from each individual system, in general, might not be large enough in size to sustain an insightful study into its individual governing principles and characteristics. Therefore, we need to aggregate all data from all systems in order to understand the systems’ fundamental dynamics. However, as we pool all individual ID-marked data sets into a huge data set, this data pooling generates many confounding effects due to existing diverse aspects of dissimilarity across all systems. Such confounding effects are collectively termed as heterogeneity. That is, the effects of governing principles are expected to interact with the effects of the heterogeneity contained in the data. This is the dilemma facing every MSP, which is the topic of this paper.

In the literature of complex system study [3,6], as well as statistical physics [7], we could not find effective approaches to coherently resolve such a dilemma in any MSPs. One chief difficulty underlying any MSP rests on the categorical nature of data in both response and covariate sides of system dynamics. Certainly, the system-ID is categorical. Another chief difficulty is heterogeneity among all member systems. These two difficulties are linked. This categorical feature naturally gives rise to multiscale issues that are closely tied to the heterogeneity potentially existing among all member systems. First, systems’ individual and distinct characteristics as fine-scale manifestations of heterogeneity can hardly be accommodated into any analytic equation or modeling structure in a systematic fashion. Secondly, the effects of interactions between the system-ID and all other features as mid-scale manifestations of heterogeneity are nearly impossible to capture precisely in man-made system descriptions. Thirdly, some individual systems are similar, while being dissimilar from other systems. Such similarity and dissimilarity among all involving systems can cause large-scale manifestations of heterogeneity.

In this paper, we first design an artificial MSP that captures the above multiscale structures and then reveals permeating effects of heterogeneity. In this example, we clearly show how heterogeneity constrains and hides essential mechanisms, and then how homogeneity can open up and reveal the true mechanisms. Then we turn to the real MSPs of interest in this paper; we consider physical pitching dynamics in Major League Baseball (MLB) in the US. A healthy starting pitcher throughout the entire season could pitch up to or even over 3000 pitches of several pitch types. Therefore, there are only several hundred pitches belonging to each pitch type for each pitcher. This amount of data is unlikely to sustain a detailed study on pitch-type specific pitching dynamics. In fact, pitching dynamics are known to be governed by the biomechanics of musculoskeletal construction and the Magnus effect of spinning baseballs in aerodynamics [8]. There are many parameters for each of these two governing principles.

Even though the biomechanics derived from musculoskeletal construction is more or less the same across all MLB pitchers, slight differences via many tuning parameters collectively make up large differences. Likewise, the Magnus effect is a universal physical phenomenon when spinning a baseball, but there are fine-scale differences in creating spins that will result in significantly distinct Magnus effects. This is why all pitchers are different. Some pitchers’ pitching dynamics are closer to those of some pitchers than others. Further, different pitch types require slightly distinct biomechanical gestures and spinning mechanisms. As such, studying a collective of pitchers’ pitching dynamics for biomechanical and physical governing principles is rather complicated. In this paper, we only focus on pitch-type specific MSPs in MLB.

In this paper, we demonstrate a computational approach for effectively studying an ensemble of pitchers’ pitch-type specific dynamic systems through two stages. In the first stage, we resolve the following essential questions. Where are the effects of heterogeneity in this MSP example? How do these effects come about? How do they interfere with the effects of governing Magnus and biomechanical effects? We explicitly explore and confirm the answers to these questions with explicit details. In a nutshell, all answers are tied to the categorical feature of system-IDs because it is highly associative with almost all response and covariate features.

In the second stage, after exploring and confirming the effects of heterogeneity, it is essential to mitigate all aforementioned effects in order to unravel critical pattern information on governing principles. To achieve this goal, ideally, we need to partition the ensemble of pitchers by grouping similar pitchers into a collection of homogeneous groups, only within which we are able to retain a large enough data set to study group-specific pitching dynamics. Consequently, the differences among these homogeneous groups are surely large-scale effects of heterogeneity.

With the resulting manifestations in the two stages, we can see and appreciate the effects of heterogeneity. These effects weave magical fabrics contained in data’s information content. This magic phenomenon mirrors what was described by Nobel laureate physicist P.W. Anderson [9] in his 1972 Science paper titled “More is Different”: from more data to more relevant scales of patterns to more surprising discoveries. In this Big Data era, MSPs are and will become even more ubiquitous in the internet. The merits of such two-stage data analysis endeavors could be critical when studying a large collection of many complex systems. We demonstrate such merits by resolving an interesting scientific problem related to the Rosenberg Self-Esteem Scale in psychology and sociology [10] by analyzing a real data set collected online.

## 2. Structural Formation and Major Factors in MSP

In this section, we postulate a generic structural formation for any MSP setting. From each single complex system member of an MSP, a data point is measured and collected in a L+KD vector format. Let the first L(=2) components be measurements or categories of the response features denoted as $Y={\left(Y_{1},\ldots ,Y_{L}\right)}^{\prime }$ and the rest of K(=10) components be measurements or categories of *K* one-dimensional covariate features denoted as $\left\{V_{1},\ldots ,V_{K}\right\}$. It is essential to note that one covariate feature, say $V_{K}$, is the categorical feature of system labels or IDs.

An unspecified complex structural relation between Y and $\left\{V_{1},\ldots ,V_{K}\right\}$ consists of a collection of *M* unknown constituent mechanisms defined by *M* major factors $\left\{F_{m}\left\{A_{m}^{*}\right\}|m=1,\ldots ,M\right\}$, in the following fashion:

(1) $$\begin{array}{cc} Y & =\left[\begin{array}{c} Y_{1}\\ Y_{2}\\ \vdots \\ Y_{L} \end{array}\right]≅G\left(F_{1}\left\{A_{1}^{*}\right\},F_{2}\left\{A_{2}^{*}\right\},\ldots ,F_{M}\left\{A_{M}^{*}\right\}\right)+\varepsilon . \end{array}$$

Here, we do not have any prior knowledge or assumptions of *M*, functional forms of $F_{m}\left\{\cdot \right\}$, the random noise ε, nor the governing structural function G(·). We only focus on identifying feature memberships of each $A_{m}^{*}\left(\subset \left\{V_{1},\ldots ,V_{K}\right\}\right)$ with m=1,…,M. By acquiring these memberships, the layouts or patterns of constituent mechanisms within dynamics underlying Y should be visible and explainable through contingency tables of $A_{m}^{*}$-vs-Y and ${⋃}_{m\in S}A_{m}^{*}$-vs-Y.

Therefore, our computational task can be simply described as: to discover the collection of major factors $\left\{A_{m}^{*}|m=1,\ldots ,M\right\}$. This computational task of major factor selection primarily relies on information theoretical measures, as will be seen in the next section.

## 3. Methods

In this section, we first briefly review the selection protocol for the major factors underlying Y as proposed and illustrated in [11]. The basic foundation is the recently developed computational paradigm called Categorical Exploratory Data Analysis (CEDA) [12,13]. The name EDA was originally coined by John Tukey [14]. The fundamental idea behind CEDA is to let all features’ natural categories assemble freely in order to shed light on the true pattern information contained in data. Then, we present a new efficient algorithm for reliability checking and a generic plan for studying any Many-System Problem.

### 3.1. Developments of CEDA

The first step of CEDA is to categorize each response and covariate feature via its histogram, which can be properly built using an effective algorithm developed in [15]. This step serves to reduce the noise inherent in all measurements in order to reveal the intrinsic categorical structure of the histogram. The second step employs a contingency table for all developments involving information theoretical measures. The contingency table is used as a platform for coupling multiple categorized features together to form and define a new composite variable. This contingency table platform also serves as a platform for visualizing and evaluating possibly non-linear associations between any two variables. The two directional associations are numerically evaluated through conditional (Shannon) entropy. By properly rescaling with respect to corresponding marginal (Shannon) entropy, a Mutual Conditional Entropy (MCE) [16] is calculated. This association measure is the correct one when considering categorical features.

The third step of CEDA computes the conditional entropies of Y given all possible covariate feature combinations or feature sets, since the structural categories of features are allowed to reassemble freely in these response-vs-covariate contingency tables without being subject to man-made constraints. This collection of all possible directional associations should ideally contain all vital associative patterns that indicate all constituent mechanisms underlying the designated response variable Y within an MSP. Our selection protocol identifies exactly such a collection of major factors. Each major factor is subject to reliability checks.

### 3.2. Major Factor Selection Based on Information Theoretical Measurements

In this subsection, we briefly review the approach to major factor selection based on the information theoretical measurements proposed in [11].

All features in Y and all covariate features, denoted as $V_{k}$ with k=1,…,K, are either categorical or categorized 1D covariate features. Let capital letters *A* or *B* denote different subsets of covariate features. Within the categorical nature of all features and the contingency table as a synthesizing platform, Y, *A*, and *B* can be treated as 1D composite categorical variables in the fashion of each occupied hypercube in their corresponding contingency table as a category.

Furthermore, any pair of 1D categorical features defines a contingency table, as do any pair of 1D composite categorical variables such as (Y,A), (Y,B), and (A,B). For a contingency table, information theoretical measurements are natural tools for discovering associative patterns. Take (Y,A) as an example. Let all categories of Y be arranged along the column-axis, while all categories of *A* are arranged along the row-axis. Then, the resultant contingency table, denoted as <Y,A>, is constructed as a rectangle array of cell counts. With suitable permutations on column- and row-axes, by aggregating unoccupied zero cells as much as possible, associative patterns and relations between Y and *A* become graphically visible. All information theoretical measurements used here are invariant with respect to row and column permutations. Using a contingency table, we can still visualize the global and large-scale pattern formations contained in <Y,A>.

The aforementioned associative patterns can in fact be numerically evaluated via various versions of conditional entropies (CEs) by basically treating <Y,A> as a 2D histogram of bivariate (Y,A). Given a column, say Y=y, we define a discrete conditional variable. Its Shannon entropy is calculated on this column’s vector of proportions, i.e., cell counts divided by its column sum, and is denoted as H[A|Y=y]. Across all rows, we calculate the weighted sum of H[Y|A=a] with respect to the weighting scheme of row sum proportions. This is the conditional entropy (CE), denoted as H[Y|A]. Likewise along the column-axis, we calculate the expected CE H[A|Y|A].

The intuitive meanings of H[A|Y] and H[Y|A] are evidently visible through their contingency tables. This CE H[Y|A] conveys the amount of expected remaining uncertainty in Y after knowing *A*. The CE drop H[Y]−H[Y|A] indicates the information explained by *A*. Therefore, it is natural to select the major factor based on the CE drop. It is worth emphasizing the fact that the conditional entropy drop indicates the shared amount information between *A* and Y; see also the review paper [17]:

$$\begin{array}{ccc} H[Y]-H[Y|A] & = & H[A]-H[A|Y]\\ & = & H[A]+H[Y]-H[A,Y]\\ & = & I[Y;A]. \end{array}$$

where H[A,Y] is the joint Shannon entropy of bivariate variable (Y,A), while I[Y;A] is the mutual information between Y and *A*.

Next, consider the CE drop of bivariate (A,B) from the CE of Y. It is shown that

H[Y]−H[Y|(A,B)]={H[Y]−H[Y|A]+H[Y]−H[Y|B]}+{I[A;B|Y]−I[A;B]}.

This simple difference {I[A;B|Y]−I[A;B]} indeed conveys the essence of interpretable meaning of conditional mutual information and plays a key role in the feature selection protocol. Considering the case of *A* and *B* being marginally stochastically independent, which means I[A;B]=0, the positivity of I[A;B|Y] indicates that the CE drop of *A* and *B* jointly is larger than the sum of the marginal ones. Similarly, if the difference {I[A;B|Y]−I[A;B]} is significantly larger than zero, this positiveness of I[A;B|Y]−I[A;B] acts like the so-called “ecological effect”; the whole is larger than the sum of its parts. The ecological effects are essential in the process of identifying a vital collection of major factors of Y, which offers an avenue for understanding mechanisms underlying Y.

On the other hand, this difference I[(A,B)|Y]−I[A;B] could be nearly zero or even negative when *A* and *B* are highly associative. In this case, at most, either *A* or *B* may be a candidate of major factors of Y, but not both. This choice of major factor is a conservative way of decision-making.

Based on the concepts above, the following two criteria, “confirmable” and “irreplaceable”, are proposed to identify a major factor of Y:

**[C1: confirmable]:**  A feature-set *A* is confirmable if a feature-set $\tilde{A}$ is obtained by substituting any one of the feature members of *A* with a feature that is completely independent of Y and *A*; we have I[Y;A] significantly larger than $I[Y;\tilde{A}]$.
**[C2: irreplaceable]:**  A feature-subset *A* is replaceable if $I\left[Y;A\right]\leq I\left[Y;A_{1}\right]+I\left[Y;A_{2}\right]$ for any compositions of *A*, i.e., $A=A_{1}⋃A_{2}$ and $A_{1}⋂A_{2}=\emptyset$. For *A* to be declared irreplaceable, we require that *A* is not replaceable and simultaneously satisfies the following two extra conditions: (a) its CE drop is larger the sum of the top ranked CE drop and at least |A|-times its complementary feature-subset CE drop; (b) the candidate *A* joined with any already identified major factor $A_{m}^{*}$ must achieve $I\left[Y;A⋃A_{m}^{*}\right]\geq I\left[Y;A\right]+I\left[Y;A_{m}^{*}\right]$.

The criterion [C1: confirmable] is mainly used as a reliability check. This is carried out by Algorithm 1.
**Algorithm 1:** Simulate a contingency table with addition of a random noise feature.
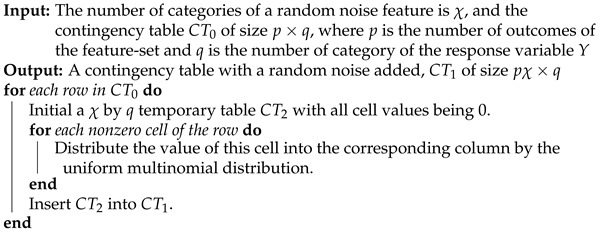


As for criterion [C2: irreplaceable], the condition (a) ensures that some kind of structural dependency among all subsets of *A* is embraced under the constraints imposed by Y, not just the occurrence of ecological effects. This condition allows the following to happen: $A_{m}^{*}\subset A_{m^{\prime }}^{*}$ for $\left(m,m^{\prime }\right)$ with $|A_{m}^{*}\left|<\right|A_{m^{\prime }}^{*}|$ and $A_{m^{\prime }}^{*}-A_{m}^{*}=B$, in the sense that *B* is the complement of $A_{m}^{*}$ in $A_{m^{\prime }}^{*}$. This selection of $A_{m^{\prime }}^{*}$ is realistic only when the CE drop of $A_{m^{\prime }}^{*}$ minus the CE drop of $A_{m}^{*}$ is many times of *B*’s CE drop. That is, $A_{m}^{*}$ and *B* have to form some “strong” bonds under the conditioning of Y in order to jointly become a higher-order major factor.

For the convenience of checking this condition, the difference between the CE drop of *A* and the top ranked CE drop of its feature-subsets is routinely calculated. It is called “successive CE drop” or denoted as “SCE drop” in tables.

The condition (b) again ensures the ecological effect among identified major factors. Though obvious, it is worth mentioning the difference between conditions (a) and (b). Condition (a) sets a very high bar for building up any high-order major factors, such as order-3 or higher, while condition (b) only requires the fulfillment of an ecological effect for any two major factors to coexist. That is, there still exists potential for a union of two identified major factors to become a high-order major factor, but condition (a) is rather hard to fulfill.

### 3.3. Reliability Checking Algorithm

To check the condition [C1: Confirmable], Algorithm 1 simulates a contingency table by expanding the covariate feature-set by including an extra random-noise feature. However, when the size of the covariate feature-set is large, the number of rows of the contingency table becomes very large. This largeness renders the algorithm inefficient. In this subsection, we propose a new algorithm to resolve this computing issue. This algorithm directly estimates the mean and variance of the conditional entropies (CEs) when expanding a covariate feature-set with an extra random-noise feature.

To achieve the goal of Algorithm 1, it suffices to estimate a CE in the case of breaking a single row into χ rows, which is the number of bins of the random-noise feature. The final CE estimate would be the weighted sum of the CE estimates across all the rows in the expanded contingency table. Consider a contingency table ${\left[n_{ij}\right]}_{\chi \times P}$ as a random matrix. Each column is a multinomial random variable with a uniform probability {1/χ,…,1/χ}. Let $n_{\cdot 1},\ldots ,n\cdot P$ be the vector of original row sums. We break each of them into χ row sums. We have

$$n_{\cdot j}=\sum_{i=1}^{\chi }n_{ij}.$$

Let sample size *n*

$$n=\sum_{j=1}^{P}n_{\cdot j}$$

and row sums $n_{i\cdot }$

$$n_{i\cdot }=\sum_{j=1}^{\chi }n_{ij}.$$

The goal is to obtain the distribution of the empirical conditional entropy $\hat{H}\left(Y|R\right)$:

$$\hat{H}\left(Y|R\right)=\sum_{i=1}^{\chi }\frac{n_{i\cdot }}{n}\sum_{j=1}^{P}\left(-1\right)\frac{n_{ij}}{n_{i\cdot }}\log\left(\frac{n_{ij}}{n_{i\cdot }}\right).$$

with some calculations below:

(2) $$\begin{array}{ccc} \hat{H}\left(Y|R\right) & = & \sum_{i=1}^{\chi }\frac{n_{i\cdot }}{n}\sum_{j=1}^{P}-\frac{n_{ij}}{n_{i\cdot }}\log\left(\frac{n_{ij}}{n_{i\cdot }}\right)\\ & = & -\frac{1}{n}\sum_{i=1}^{\chi }\sum_{j=1}^{P}n_{ij}\log\left(\frac{n_{ij}}{n_{i\cdot }}\right)\\ & = & -\frac{1}{n}\sum_{i=1}^{\chi }\sum_{j=1}^{P}n_{ij}\log\left(n_{ij}\right)+\frac{1}{n}\sum_{i=1}^{\chi }\sum_{j=1}^{P}n_{ij}\log\left(n_{i\cdot }\right)\\ & = & -\frac{1}{n}\sum_{j=1}^{P}\sum_{i=1}^{\chi }n_{ij}\log\left(n_{ij}\right)+\frac{1}{n}\sum_{i=1}^{\chi }n_{i\cdot }\log\left(n_{i\cdot }\right)\\ & = & \left[\sum_{j=1}^{P}\frac{n_{\cdot j}}{n}\sum_{i=1}^{\chi }-\frac{n_{ij}}{n_{\cdot j}}\log\left(\frac{n_{ij}}{n_{\cdot j}}\right)\right]-\sum_{j=1}^{P}\frac{n_{\cdot j}}{n}\log\left(\frac{n_{\cdot j}}{n}\right)+\sum_{i=1}^{\chi }\frac{n_{i\cdot }}{n}\log\left(\frac{n_{i\cdot }}{n}\right) \end{array}$$

The first term of the last equation, $\sum_{j=1}^{P}\frac{n_{\cdot j}}{n}\sum_{i=1}^{\chi }-\frac{n_{ij}}{n_{\cdot j}}\log\left(\frac{n_{ij}}{n_{\cdot j}}\right)$ can be viewed as the weighted empirical conditional entropy $\sum_{j=1}^{P}\frac{n_{\cdot j}}{n}\hat{H}\left(multi\left(n_{\cdot j},\left[\frac{1}{\chi },\ldots ,\frac{1}{\chi }\right]\right)\right)$. The second term $-\sum_{j=1}^{P}\frac{n_{\cdot j}}{n}\log\left(\frac{n_{\cdot j}}{n}\right)$ is the empirical conditional entropy of $\left\{n_{\cdot 1},\ldots ,n_{\cdot P}\right\}$, which is a fixed number. The last term $\sum_{i=1}^{\chi }\frac{n_{i\cdot }}{n}\log\left(\frac{n_{i\cdot }}{n}\right)$ is the empirical conditional entropy $-\hat{H}\left(multi\left(n,\left[\frac{1}{\chi },\ldots ,\frac{1}{\chi }\right]\right)\right)$.

In practice, it is easier to use (2) to understand $\hat{H}\left(Y|R\right)$. The mean of $\hat{H}\left(Y|R\right)$ equals the mean of $\sum_{j=1}^{P}\sum_{i=1}^{\chi }n_{ij}\log\left(n_{ij}\right)+\frac{1}{n}\sum_{i=1}^{\chi }n_{i\cdot }\log\left(n_{i\cdot }\right)$, where each term can be numerically computed separately. For a positive integer *N*, let $x=(x_{1},\ldots ,x_{\chi })$ be the random variable from $multi(N,\left[\frac{1}{\chi },\ldots ,\frac{1}{\chi }\right])$. Define a function h(x) as

$$h\left(x\right)=\sum_{i=1}^{\chi }x_{i}\log\left(x_{i}\right).$$

We can numerically compute the mean and variance of h(x). Denote m(N) and v(N) as the mean of h(x) for *x* from $multi(N,\left[\frac{1}{\chi },\ldots ,\frac{1}{\chi }\right])$. From (2), we have

(3) $$E\left[\hat{H}\left(Y|R\right)\right]=\frac{1}{n}\left[m\left(N\right)-\sum_{j=1}^{P}m\left(n_{\cdot j}\right)\right].$$

For the variance, there is no exact and simple form, since the row sums $n_{i\cdot }$’s are dependent on columns. However, since the entropy of the row sums is positively correlated to the sum of the entropies of the columns, it is safe to have $\sum_{j=1}^{P}v\left(n_{\cdot j}\right)/n^{2}$ as the upper bound of the variance of $\hat{H}\left(Y|R\right)$:

(4) $$Var\left[\hat{H}\left(Y|R\right)\right]\leq \frac{1}{n^{2}}\sum_{j=1}^{P}v\left(n_{\cdot j}\right).$$

In practice, when there are many rows in a contingency table, the number in each cell of the contingency table will not be large. That is, the unique numbers in the contingency table are relatively few. Therefore, it will become efficient to estimate the mean and variance using (3) and (4). Here we remark that the estimate of the mean by (3) is in fact more accurate than that from Algorithm 2, and the variance estimation is usually within 1.2 times the true value from our experimental experience.
**Algorithm 2:** Estimating the mean and the variance of the conditional entropy with a random noise feature included.
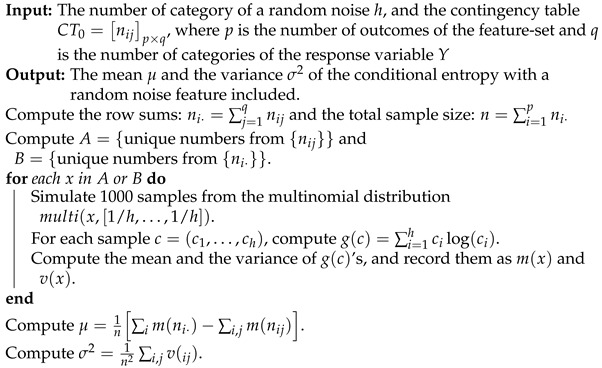


### 3.4. A Generic Plan for Studying Any Many-System Problem

How can we effectively deal with complexity and heterogeneity embedded within any MSP? We propose the following study plan for MSPs under a framework with a designated response variable Y and a collection of covariate features $\left\{V_{1},\ldots ,V_{K-1}\right\}$ across all the individual systems.

**MSP-1:**  Make the system-ID into an extra categorical feature and denote it as $V_{K}$;
**MSP-2:**  Treat the MSP as a system described by Equation (1);
**MSP-3:**  Perform the CEDA-based feature selection protocol to identify a vital collection of major factors, each of which is a feature-subset of $\left\{V_{1},\ldots ,V_{K}\right\}$ that satisfies both criteria [C1: confirmable] and [C2: irreplaceable];
**MSP-4:**  Apply the hierarchical clustering algorithm to partition the collection of systems into homogeneous system-clusters. This clustering is undertaken based on measurement vectors of major factors without including $V_{K}$.

Here, a homogeneous system-cluster means that we find no major factors containing $V_{K}$ when a system-cluster is treated and studied as a small version of MSP by going through the steps MSP-1 to MSP-3. The collection of homogeneous system-clusters explicitly reveals the systemwise heterogeneity. Through computational results of MSP-2 and MSP-3, we will evidently see the interacting relationships between complexity and heterogeneity through memberships of major factors within the original MSP under study.

The above study plan for MSPs is proposed based on experience. As expected, and as will be demonstrated in the MLB examples, the complexity and heterogeneity of any MSP interact with each other in many unknown ways across multiple scales. Recognizing this leads us to adopt Equation (1) by having all the functional structures be completely unspecified and unknown. Without knowing the global functional structure G(.) and the component-wise structures $\left\{F_{m}\left(.\right)|m=1,\ldots ,M\right\}$, our feature selection protocol can freely discover “major factors of various orders” by being free from man-made structural constraints. It is worth reiterating that this study plan works equally well for all data types.

Being free from structural and data-type constraints is critical and essential to understand and resolve real-world problems reliably. That is, we can not only build understanding of any complex system with a large amount of available data, but also realistically resolve MSPs in their original forms with reliability. Especially in this Big Data era, nearly all researchers admit that no modeling can stand true when faced with large amounts of data. Although the phrase: “All models are wrong, but some are more useful than others”, still rings true, we do have one viable alternative: CEDA-based selection for major factors.

Certainly, such freedom of functional structures and universal validity for all data types come at the cost of facing the “finite sample phenomenon”, since reliable contingency tables can only be constructed with a limited number of covariate features, which mirror the “curse of dimensionality”. Such a cost could result in missing high-order major factors in MSPs. However, all data sets are finite. On the other hand, high-order major factors are harder to confirm. Nevertheless, vital and reliable collections of low- and median-order major factors constitute authentic intelligence when they are fully supported by visible and explainable patterns using contingency table platforms.

## 4. A Designed MSP Example

In this section, we design an MSP to demonstrate the complicated effects of heterogeneity. It is intuitive that such effects are especially profound when many complex systems are involved within an MSP, since a large number of constituent systems means that data points within each category of response features are spread thinner across all system-IDs, coupled with other covariate features. Consequently, all conditional entropy computations within such an MSP study are prone to the finite sample phenomenon. With such intuition in mind, we design our experimental MSP as follows.

Let the $V_{1}$ be a categorical feature with 80 categorical-IDs digital coded as: 1 to 80. These 80 ID-codes are equally divided into eight homogeneous groups defined as follows:

$G_{1}$. $Y=V_{2}+sin\left(2\pi \times \left(V_{4}+V_{5}+V_{6}\right)\right)/2+N\left(0,1\right)/10$,
$G_{2}$. $Y=V_{2}+sin\left(2\pi \times \left(V_{3}+V_{5}+V_{6}\right)\right)/2+N\left(0,1\right)/10$,
$G_{3}$. $Y=V_{2}+sin\left(2\pi \times \left(V_{3}+V_{4}+V_{6}\right)\right)/2+N\left(0,1\right)/10$,
$G_{4}$. $Y=V_{2}+sin\left(2\pi \times \left(V_{3}+V_{4}+V_{5}\right)\right)/2+N\left(0,1\right)/10$,
$G_{5}$. $Y=V_{2}+sin\left(2\pi \times U^{\left(5\right)}\left[0,1\right]\right)/2+N\left(0,1\right)/10$,
$G_{6}$. $Y=V_{2}+sin\left(2\pi \times U^{\left(6\right)}\left[0,1\right]\right)/2+N\left(0,1\right)/10$,
$G_{7}$. $Y=V_{2}+sin\left(2\pi \times U^{\left(7\right)}\left[0,1\right]\right)/2+N\left(0,1\right)/10$,
$G_{8}$. $Y=V_{2}+sin\left(2\pi \times U^{\left(8\right)}\left[0,1\right]\right)/2+N\left(0,1\right)/10$,

where, beside $V_{1}$, all 11 covariate features $\left\{V_{2},\ldots ,V_{12}\right\}$ are mutually independent and distributed according to Uniform[0,1]. There are the four unobserved hidden covariates $\left\{U^{\left(k\right)}\left[0,1\right]|k=5,\ldots ,8\right\}$ contained in $G_{5}$ through $G_{8}$. That is, the six observed covariate features $\left\{V_{7},\ldots ,V_{12}\right\}$ have nothing to do with the response variable *Y* and are independent of other covariate features.

For each ID via $V_{1}$, we simulate 500 $\left(Y,V_{2},\ldots ,V_{12}\right)$ data points. Each group contains 10 IDs, so there are 5000 data points for each of the eight groups. Based on the whole set of 40,000 data points, we compute all conditional entropy of *Y* given all possible feature subsets of $\left\{V_{1},\ldots ,V_{12}\right\}$. In particular, we also compute and report the successive conditional entropy (SCE) drops of such feature subsets. Here we reiterate once more, for a feature-set *A*, its SCE drop is defined as: CE drop of *A* (H[Y]−H[Y|A]) minus the maximum of CE drops of all subsets of *A*. We report SCEs in Table 1 across one-feature to three-feature settings. All feature-sets within all settings with more than three features do not meet criterion [C1:confirmable]. We summarize the results derived from our major factor selection protocol as follows.

Selection of Major Factors in the Designed MSP

**1**  In the one-feature setting of Table 1, $V_{2}$ achieves the smallest CE with the largest CE drop: 0.3332. We surely recognize $V_{2}$ as the potential candidate for order-1 major factor. In contrast, $V_{1}$ achieves the second ranked CE with a CE drop of 0.0106, which is significantly less than that of $V_{2}$, but it is almost 10 times the CE drop of any of the rest of the 10 features, including the four features $\left\{V_{3},\ldots ,V_{6}\right\}$ involving $G_{1}$ through $G_{4}$, all of which achieve tiny CE drops around 0.001. In fact, $V_{1}$ does not meet criterion [C1: confirmable], since its CE, 2.4231, is beyond the center of CE distribution of ε (having 80 categories) with mean 2.4229 and $sd=5.2654\times 10^{-4}$. Therefore, $V_{1}$ is not a candidate of order-1 major factor. Furthermore, no individual feature of $\left\{V_{3},\ldots ,V_{12}\right\}$ meets the criterion [C1: confirmable].
**2**  In the two-feature setting, we can see that the first 11 pairs of the 12 ranked SCEs are made of $V_{1}$ coupling with $\left\{V_{2},\ldots ,V_{12}\right\}$, and their SCEs are all many times larger than their corresponding individual CE drops. Therefore, all these feature-pairs satisfy the criterion [C2: irreplaceable]. However, even the top four pairs, $V_{1}\_V_{3}$, $V_{1}\_V_{4}$, $V_{1}\_V_{5}$ and $V_{1}\_V_{6}$, do not meet criterion [C1: confirmable]. Through Algorithm 2, the distribution of CEs of $\left(V_{1},\varepsilon \right)$ is found to have a mean of 2.2894 and an SD of 0.0019. The CE of $V_{1}\_V_{3}$, 2.2895, is slightly larger than the mean. This is partly due to the fact that there are 80 categories of $V_{1}$. Thus, there are no confirmed candidates of order-2 major factors. It is notable that the pair $\left(V_{1},V_{2}\right)$ achieves a SCE of 0.1035, which is about 10 times as large as the CE drop achieved by $V_{1}$. That is, by satisfying the ecological effect, $V_{2}$ and $V_{1}$ can be order-1 major factors simultaneously.
**3**  In the three-feature setting, the top six feature triplets are $V_{1}$ coupling any pairs of $\left\{V_{3},V_{4},V_{5},V_{6}\right\}$. They achieve SCEs that satisfy criterion [C2: irreplaceable], but they do not fulfill the criterion [C1: confirmable]. The CEs of these triplets, which range between 1.2146 and 1.2198, are all larger than the mean (1.2051) of the distribution of the CEs of $\left(V_{1},V_{3},\varepsilon \right)$. Therefore, there are no confirmed candidates for order-3 major factors.
**4**  In the four-feature setting, the four feature quartets, $V_{1}$ coupling any triplets of $\left\{V_{3},V_{4},V_{5},V_{6}\right\}$, do not meet the criterion [C1: confirmable].

From the above summary, it becomes evident that the effects of heterogeneity of $V_{1}$ permeate and blur candidacies of order-2 and order-3 major factors. Such blurred pictures are primarily due to the finite sample phenomenon. That is, the heterogeneity causes the loss of full views of the true underlying mechanisms. Accordingly, based on results from our major factor selection protocol, we conclude that the chief collection of major factors is $\left\{V_{2}\right\}$ and there are no alternative collections.

**Figure 1:**
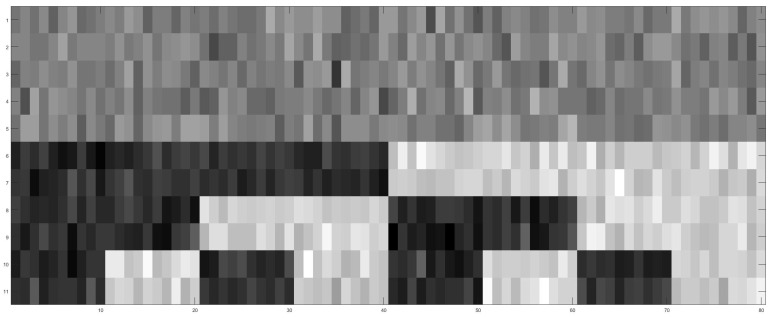
Clustering on 80 IDs’ vectors of means of 11 features.

Next, we proceed with the decomposition of the whole data set’s heterogeneity into homogeneous parts. Indeed, as shown in Figure 1, G1 to G8 are correctly identified on a heatmap clustering 80IDs’ vectors of means pertaining to the 11 features. When the exclusive data of $G_{1}$ through $G_{8}$ are analyzed separately, we can precisely confirm major factors of various orders underlying each of the eight groups. The order-1 major factor $V_{2}$ is confirmed, but no candidates of order-2 major factors are confirmed across the eight groups. As for potential candidate order-3 major factors, for instance in $G_{1}$, the feature-triplet $\left(V_{4},V_{5},V_{6}\right)$ achieves a CE of 1.0432, satisfying the criterion [C2: irreplaceable], and meets [C1: confirmable] in the following fashion. By applying Algorithm 2, this triplet’s CE is more than 10 SD below the CE distribution of $\left(V_{4},V_{5},\varepsilon \right)$ with mean 1.1094 and SD 0.0058, where ε is a uniform noise feature. Likewise, we confirm the order-3 major factors in $G_{2}$ through $G_{4}$, respectively. We summarize the results of our major factor selection within this designed MSP in Table 2. It is noted that the results of $G_{6}$, $G_{7}$, and $G_{8}$ are identical to those of $G_{5}$.

## 5. MLB’s Two MSPs: Sliders and Fastballs

In this section, we study Major League Baseball (MLB) pitching dynamics from an MSP perspective.

MLB owns two databases, namely PITCHf/x and Statcast, that record every pitch delivered in all MLB games since 2006. In this paper, we study two large real-world MSPs of slider and fastball pitching dynamics in the 2017 season. From the PITCHf/x database, we select all pitchers who have pitched more than 500 sliders or fastballs, respectively. There are 62 slider pitchers and 199 fastball pitchers that meet this 500 pitches criterion. The pitch-ID is denoted as pitN.

In a structured data format, biomechanical and physical features include a pitched baseball’s releasing coordinates {x0,z0}, speeds {vX0,vY0,vZ0,}, and accelerations {aX,aY,aZ} along the horizontal (X−), vertical (Z−), and pitcher-to-catcher (Y−) directions. The feature of starting speed denoted by startSp is very close to vY0. Two features are measured for each pitch’s spin information: spin direction spinD and spin rate spinR. The aforementioned features are the covariate features of pitching dynamics measured around the pitcher mound, while the two features measured around the home plate, horizontal and vertical movements denoted by $pfx_{x}$ and $pfx_{z}$, are designated as a 2D response variable $Y=(pfx_{x},pfx_{z})$. In both pitch-type specific MSPs, each pitching dynamic of $\left(pfx_{x},pfx_{z}\right)$ is found to be dominated by (aX,aZ) under the presence of pitN. Therefore, we use (aX,aZ)=Y as a 2D response variable against the remaining covariate features to further explore the effects of pitN. All aforementioned features, except pitN, are quantitative and undergo categorization. All categorized features retain the same names throughout this paper.

MLB pitching dynamics are basically governed by biomechanical forces in the X,Y, and *Z* directions, coupled with the Magnus effect of the “spinning baseball” created by the friction between the pitcher’s fingers and the baseball surface, and the gravity of earth. The Magnus effect is a 3D force constantly acting on the baseball with a 2D direction on the 2D disk that is perpendicular to the baseball’s trajectory at all time. Its 2D direction is measured by spin direction (spinD), while its stability is determined by spin rate (spinR) and various directional speeds, accelerations, and other elements. It is not an easy task to clearly separate the force due to the Magnus effect from the biomechanical ones in pitching dynamics.

Different pitch types require different kinds of spinning. Back-spinning in fastballs persistently shows four or two seams rotating backward when viewed from the catcher or batter’s perspective. This is a natural type of spin that can be easily created. Its Magnus effect points upward against gravity. The top-spinning in curveballs shows seams rotating forward instead. Its Magnus effect points downward to the ground and adds to gravity. Sliders are slower than fastballs, but much faster than curveballs in speed. Their spinning direction has a much wider range than fastballs and the curveballs. That is, the Magnus effect makes sliders have wider ranges of vertical movement (measured and denoted by $pfx_{Z}$) and horizontal movement (measured and denoted by $pfx_{X}$) than that of fastballs and curveballs.

These two measurements measured around the home plate are critical to pitchers when trying to effectively deal with batters. Therefore, we first designate $\left(pfx_{Z},pfx_{X}\right)$ as the 2D response variable. Then, the MSP of interest can be very precisely depicted as the fundamental question: what are the major factors underlying $\left(pfx_{Z},pfx_{X}\right)$ among many pitchers? This description does not mean that the identified collection of major factors would manifest and separate Magnus effects and biomechanical forces in a clear-cut fashion. On the contrary, the collective of major factors only reveals major factors that most directly cause the designated response variable. That is, major factors are very sensitive to the choice of response variables.

For the two focal pitch types in the 2017 MLB season, there are 62 slider pitchers and 199 fastball pitchers who pass the 500 pitches threshold. We have a 2D response variable $Y=(pfx_{Z},pfx_{X})$ and 12 covariate features that all are measured around the pitcher’s mound.

### 5.1. Sliders MSP

**Figure 2:**
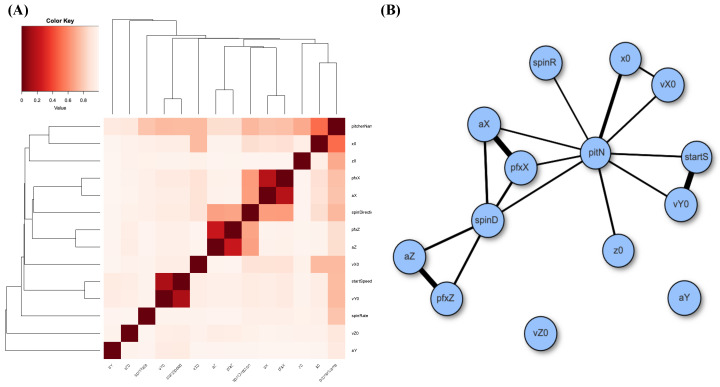
Associative patterns of sliders among 14 features: (**A**) heatmap based on MCE-matrix; (**B**) network built with linkages with thickness proportional to one minus pairwise MCE and subject to a threshold 0.2 (=1.0−MCE).

A heatmap and a network of MCE-based associative patterns of these 14 features, responses, and covariates are given in panels (A) and (B) of Figure 2. It is clear that feature-pairs $\left(pfx_{X},aX\right)$ and $\left(pfx_{Z},aZ\right)$ are highly associative, while aX and aZ are not. Based on the heatmap and network, we clearly see diverse associative patterns among these 14 features.

We computed conditional entropies for all possible combinations of 12 covariate features. The resultant table is too large to be included entirely. Thus, we report only top-ranked CEs up to five-feature setting in Table 3 and SCE drops up to four-feature setting in Table 4. These two formats of tables are used throughout the three MLB-related MSPs for expositional efficiency of showing potential candidates of major factors. However, our search for feature selection of major factors is conducted based on all possible feature-sets. We summarize patterns from our feature selection protocol and list our reasoning based on the criteria [C1:confirmable] and [C2:irreplaceable] across all feature settings. The CE of Y is calculated as 4.6890.

**Figure 3:**
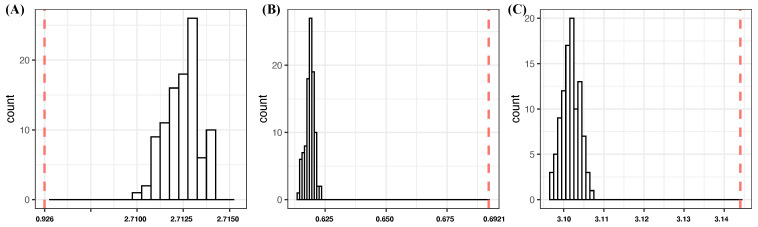
Testing for [C1: confirmable] on $Y=(pfx_{X},pfx_{Z})$: (**A**) (aX,aZ) (red-marked) against histogram of (aX,ξ); (**B**) (aX,aZ,pitN) and (aX,aZ,vY0) (red-marked) against histogram of (aX,aZ,ξ); (**C**) (vZ0,z0,aY) (red-marked) against histogram of (vZ0,z0,ξ).

**1:**  In the one-feature setting, we see four features—{spinD,aX,pitN,aZ}—that achieve CE drops more than 1.1000. They are potential candidates of order-1 major factors. Among these four features, {spinD,aX,pitN} are moderately associated based on Figure 2A,B. This fact would significantly reduce their potentials to simultaneously be order-1 major factors.
**2:**  Considering the two-feature setting, the feature-pair (aX,aZ) achieves the lowest CE, 0.9264, and meets the criterion [C1: confirmable], see Figure 3A. However, its CE drop, 3.7626, is almost equal to the sum of individual CE drops of aX and aZ: 3.8032. Even though, based on Figure 2A,B, aX and aZ are not moderately dependent to each other, this observation of I[aX;aZ|Y]≤I[aX;aZ] strongly indicates that both can not be order-1 major factors simultaneously. Certainly, the feature-pair (aX,aZ) is not an order-2 major factor because of the criterion [C2:irreplaceable]. In fact, the rest of five feature-pairs derived from {spinD,aX,pitN,aZ} share the same situations, such as (aX,aZ).
  Four feature-pairs (aY,pitN), (z0,pitN), (spinR,pitN) and (vZ0,pitN), which are formed by coupling pitN with one member of feature-set{aY,vZ0,spinR,z0}, are potential candidates for order-2 major factors. In fact, they meet the criterion [C1:confirmable], and their SCE drops from pitN, respectively, are six and three times the individual CE drops of aY, z0, spinR, and vZ0. We also find the 10 pairs {aY,z0}×{x0,vY0,vX0,vZ0,spinR} and (aY,z0) are also candidates of order-2 major factors, satisfying the two criteria. Here we use the threshold of “3 times” in condition (a) of criterion [C2:irreplaceable], while the two pairs (spinR,vY0) and (spinR,startSp) are excluded, since their SCE drops are 0.3788 and 0.3772 from either vY0 or spartSp, which are at most 2.5 times the individual CE drop of spinR.
  If we accept any of these 15 as order-2 major factors, then we consequently decide which one {spinD,aX,pitN,aZ} is an order-1 major factor subject to the criterion [C2:irreplaceable]. For example, if we pick (aY,vZ0) as an order-2 major factor, then only aX, aZ, and pitN are qualified to be order-1 major factors. The collections {aX,(aY,vZ0)} and {aZ,(aY,vZ0)} achieve CE values 2.2452 and 2.3466, respectively. Both collections meet the [C1:confirmable] criterion.
  In contrast, it is interesting and important to note that, though the collection {pitN,(aY,vZ0)} achieves a CE value 1.9449, which is smaller than the aforementioned collections, it does not meet the [C1:confirmable] criterion. By applying Algorithm 2, the simulated CE distribution of (pitN,vZ0,ξ) has a mean value 1.7586 and an SD of $1.9830\times 10^{-3}$. The underlying reason is the finite sample phenomenon caused by the 62 categories of pitN. The effect of this finite sample phenomenon becomes less when the ensemble of 62 pitchers is partitioned into groups.
  If we pick (vY0,z0) as an order-2 major factor, then only aZ and pitN are qualified to be order-1 major factors. The collection {aZ,(vY0,z0)} achieves a CE value 2.1633, while the collection {pitN,(vY0,z0)} achieves a CE value 2.6462.
  If we pick either (vZ0,z0) or (spinR,z0) as an order-2 major factor, then aX, aZ, pitN, and spinD are qualified to be order-1 major factors. The two collections {aX,(vZ0,z0)} and {aZ,(spinR,z0)}, respectively, achieve the smallest CE values of 2.2224 and 2.2534 within the two packs of the four collections.
**3:**  In the 3-feature setting, the top 10 feature triplets have achieved rather uniform CEs, while the 12 ranked triplets have rather uniform SCE drops. Such uniformness is likely a sign of the finite sample phenomenon. Hence, it becomes necessary to test whether our feature selection should stop before the three-feature setting. This testing is first performed on the triplet (aZ,vY0,aX). It indeed meets the [C1:confirmable] criterion; see panel (B) of Figure 3. However, it is far from meeting the criterion [C2:irreplaceable].
  As for triplet (aY,vZ0,pitN), it achieves the largest SCE drop and seemingly has potential to be order-3 major factor. However, it is not, due to the criterion [C2:irreplaceable]. The reason is this is that triplet (aY,vZ0,pitN) is a union of (aY,pitN) and (vZ0,pitN) with individual CE drops of 1.5820 and 1.5908, respectively. The sum of both CE drops, 3.1728, is much larger than the CE drop of the triplet: 2.7441(=4.6890−1.9449). Despite this, as indicated above, the collection {pitN,(aY,vZ0)} is a valid one. That is, it takes much more to become a high order major factor.
  In contrast, the remaining 11 triplets achieve rank 2 to 12 successive CE drops in Table 4, such as {aY,vZ0,z0}, do not meet the criterion [C1:confirmable]; see Figure 3C. In conclusion, there are no order-3 major factors confirmed.
**4:**  In four-feature setting, the top two quartets (aZ,aY,aX,pitN) and (aZ,vZ0,aX,pitN) achieve the lowest CE. They consist of three potential order-1 major factors, aX, aZ, pitN, so they do not meet the criterion [C2:irreplaceable]. Similar reasoning is applicable to the quartets (aZ,vY0,aX,pitN), (aZ,startSp,aX,pitN), (aZ,aY,aX,startSp), and (aZ,z0,aX,vY0).
**5:**  In the 5-, 6- and 7-feature settings, they fail to meet the criterion [C1:confirmable] due to the finite sample phenomenon. For instance, if we collect members of confirmed potential major factors of all orders, we arrive at a seven-feature set (aZ,aX,vY0,vZ0,pitN,spinR,aY). THis indeed achieves the lowest CE in the seven-feature setting. However, it does not meet the criterion [C1:confirmable], since, according to Algorithm 2, its CE of 0.0236 is far beyond the range of the simulated CE distribution of (aZ,aX,vY0,vZ0,pitN,spinR,ξ), with mean $8.9602\times 10^{-3}$ and SD $5.8219\times 10^{-4}$.

From the above feature-selection, we identify {aZ,(vY0,z0)} as the chief collection of major factors of $Y=(pfx_{X},pfx_{Z})$, and two alternative collections: {aX,(vZ0,z0)} and {aX,(vZ0,aY)}. These three collections contain one order-1 and one order-2 major factor. We found no order-3 or higher orders. Each order-1 major factor, either aZ or aX, within any of these three collections contributes significantly more than its corresponding order-2 major factor. If the fourth alternative collection is needed, then it would be {aZ,(spinR,z0)}.

It is also evident that the effect of heterogeneity is not explicitly present because of pitN is not selected as an order-1 major factor, while the Magnus effect is also not visible, since spinD is not selected as one of order-1 major factors in the three selected collections. However, collections such as {spinD,(vZ0,z0)} and {spinD,(spinR,z0)} are not selected because their CE values are much higher than the selected ones.

Finally, all these collections of major factors of $Y=(pfx_{X},pfx_{Z})$ involve two features of four aspects of the pitching dynamics: (1) the acceleration in the *Y*-direction (aY), (2) the vertical releasing speed (vZ0), (3) spin rate spinR, (4) the vertical coordinate of the releasing point z0. This fact collectively indicates that our understanding of the underlying dynamics of $Y=(pfx_{X},pfx_{Z})$ should be derived from multiple perspectives. It is also evident that the effect of spinR is not ignorable; nonetheless, it is definitely not the primary effect.

On the other hand, aX and aZ seem to play key roles in $Y=(pfx_{X},pfx_{Z})$. This pair achieves a CE drop of 3.7626 from a CE of Y: 4.6890. In contrast, the chief and alternative collections of major factors of $Y=(pfx_{X},pfx_{Z})$ have CE drops of about or less than 2.6890. That is, we need to further investigate by using Y=(aX,aZ) for the 25% unexplained CE beyond our collections of major factors.

Another motivation for looking at the dynamics of Y=(aX,aZ) is the fact that the significance of the Magnus effect is not evidently reflected in the collections of identified major factors of $Y=(pfx_{X},pfx_{Z})$. In fact, the spin direction (spinD) is not a primary member of the identified collections of major factors. Obvious reasons might be that this 2D response variable is highly and directly associated with aX, aZ and the heterogeneous effect via pitN. That is, the Magnus effect and the heterogeneity effect have likely been overshadowed by aX and aZ. To further investigate this speculation, we need to conduct another study via feature selection for major factors of response variable Y=(aX,aZ).

There are 10 covariate features for this investigation in the same MSP of 62 slider pitchers. Likewise we compute all CEs for all possible combinations of 10 features. We report up top 10 CE values across five feature-settings in Table 5 and the top 10 SCE drops in Table 6 across four feature-settings. Computational results of feature selection for major factors are summarized below starting from one-feature setting to five-feature setting. The CE of Y is 4.7213.

**1**  In the one-feature setting, spinD and pitN achieve the top two lowest CEs. Their CE drops, 1.6912 and 1.1127, respectively, are many time larger than the rest of eight features’ CE drops. However, we do not expect them to become two separate order-1 major factors due to their moderate mutual association. This can be seen through the fact that the feature-pair (spinD,pitN) achieves a SCE drop of 0.5410 from spinD, which is less than half the CE drop of pitN. Again, the selection of an order-1 major factor is highly dependent on the selection of order-2 major factors.
**2**  Considering the two-feature setting, the three feature-pairs: pitN coupled with one member of feature-set {aY,vZ0,z0} have SCE drops at least three times the CE drop of aY, z0, and vZ0, respectively. They satisfy the criterion [C2: irreplaceable] and meet the criterion [C1:confirmable]. We also have 10 pairs, {aY,z0}×{x0,vY0,vX0,vZ0,spinR}, and (aY,z0), that fulfill the two criteria. They are potential candidates for order-2 major factors. Any of them could become an order-2 major factors depending on which order-1 major factor is selected.
  With respect to all order-1 and order-2 candidates for major factors, we select the collection {spinD,(vZ0,z0)} as the chief collection of major factors of Y=(aX,aZ). We also select two alternative collections:{spinD,(aY,vZ0)} and {spinD,(aY,z0)}. All candidate collections involve pitN, and the aforementioned candidates of order-2 major factors can not meet the criterion [C1:confirmable] under the three-feature setting.
**3**  In the three-feature setting, all top 10 triplets of CEs involve pitN, while seven out of ten involve the spinD. These 10 triplets are not the top 10 of CE drops and they achieve rather uniform CEs. According to Algorithm 2, they all fail to meet the criterion [C1:confirmable]. For example, the triplet (vZ0,pitN,aY) achieves the lowest CE of 1.9577 in this three-feature setting. This CE value is far beyond the range of the simulated CE distribution of (vZ0,pitN,ξ), with mean 1.7702 and SD $1.9173\times 10^{-3}$.
**4**  Regarding the four- and five-settings, they achieve rather uniform CEs. This is a sign of the effect of the finite sample phenomenon. That is, based on Algorithm 2, they all fail to meet the criterion [C1:confirmable]. For example, (vZ0,spinR,pitN,aY) has a CE of 0.9624, far beyond the range of the simulated CE distribution of (vZ0,spinR,pitN,ξ), with mean 0.7718 and SD $1.172166\times 10^{-3}$. Furthermore, (vY0,vZ0,spinR,pitN,aY) has a CE of 0.3837, far beyond the range of the simulated CE distribution of (vY0,vZ0,spinR,pitN,ξ), with mean 0.2649 and SD $7.378123\times 10^{-4}$.

Our conclusion in this investigation where Y=(ax,az) is that the fact that the three collections have spinD as the order-1 major factor is rather natural based on general knowledge of pitching dynamics. The three members of order-2 major factors are pairs from {z0,aY,vZ0}. That is, the biomechanical features do play some important roles underlying the dynamics of Y=(ax,az). Further, the effects of heterogeneity within this collective of 62 slider pitchers systems seem evidently overshadowed by the finite sample phenomenon in the three-feature setting and beyond, though these effects are reflected through presences of multiple candidates of order-2 major factors.

### 5.2. Fastballs: 199 Pitchers

**Figure 4:**
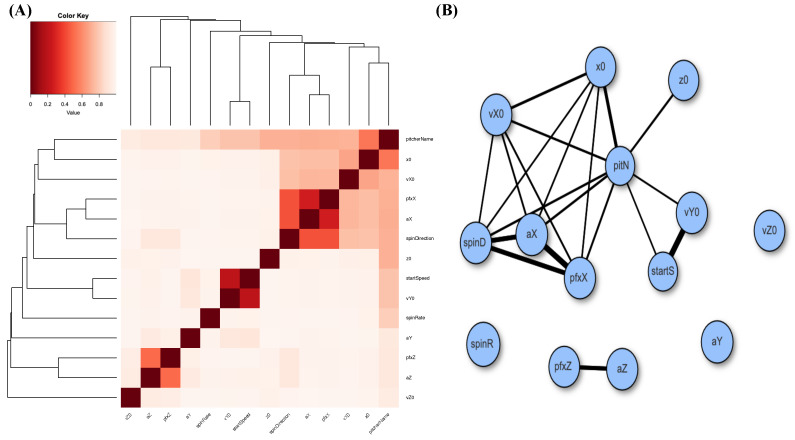
Associative patterns of fastballs among 14 features: (**A**) heatmap based on MCE-matrix; (**B**) network built with linkages with thickness proportional to one minus pairwise MCE and subject to a threshold 0.2 (=1.0−MCE).

The MSP of fastballs in the 2017 MLB season consists of 199 pitchers with 14 features. In the two panels of Figure 4, the heatmap and the network based on the 14×14 MCE matrix reveal again that feature-pairs $\left(pfx_{X},aX\right)$ and $\left(pfx_{Z},aZ\right)$ are highly associative, but that the pair (aX,aZ) is not.

Again, we first consider the 2D bivariate response variable $Y=(pfx_{X},pfx_{Z})$. The CE of Y is calculated as 4.7167. According to the two criteria [C1: confirmable] and [C2: irreplaceable], we summarize our feature selection for major factors of $Y=(pfx_{X},pfx_{Z})$ based on the CEs of all the possible feature-sets from the 12 covariate features. Only the top ranked CEs and SCE drops are reported in Table 7 and Table 8.

**Figure 5:**
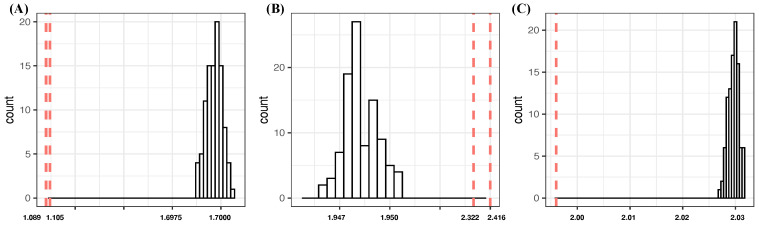
Testing for [C1: confirmable] on $Y=(pfx_{X},pfx_{Z})$: (**A**) (aX,aZ,vY0) and (aX,aZ,startSp) red-marked against histogram of (aX,aZ,ε); (**B**) (aY,pitN,spinR) and (aY,pitN,startSp) red-marked against histogram of (aY,pitN,ε); on Y=(ax,az): (**C**) (spinD,pitN,aY) red-marked against histogram of (spinD,pitN,ε).

**1:**  With regard to the one-feature setting, we see four features, {spinD,aX,pitN,aZ}, that achieve CE drops larger than 1.2000. They are clearly potential candidates for order-1 major factors, since spinD and aX are highly associated and both are moderately associated with pitN. Such associative relations among these three features keeps them from being order-1 major factors simultaneously. In contrast, aZ is a strong potential candidate for an order-1 major factor.
**2:**  In the two-feature setting, the feature-pair (aX,aZ) achieves the lowest CE of 1.7424, with a CE drop of 2.9763, which is almost equal to the sum of the individual CE drops of aX and aZ: 2.9197; that is, I[aX;aZ|Y]≃I[aX;aZ] . Together with the fact that, based on Figure 4A,B, aX and aZ are independent; only one of them can be an order-1 major factor in a collection of major factors. We also identify six feature-pairs, (aZ,vY0), (aY,vY0), (z0,vY0), (vZ0,pitN), (vY0,pitN), and (aY,pitN), that satisfy the two criteria [C1: confirmable] and [C2: irreplaceable].
  Therefore, we select the chief collection {aX,(vZ0,aZ)} because it achieves the lowest CE value in the three-feature setting, and just barely satisfies the criterion [C2: irreplaceable]. Two alternative collections are: {aZ,(vY0,aY)} and {aZ,(vY0,z0)}. On the other hand, collections {aZ,(vZ0,pitN)}, {aZ,(vY0,pitN)}, and {aZ,(aY,pitN)} fail to meet the criterion [C1: confirmable].
**3:**  In the three-feature setting, the triplets (aX,aZ,vY0) and (aX,aZ,startSp) (see Figure 5A pass the test of [C1:confirmable]. They achieve the top two ranked CEs with their CE drops of 0.6536 and 0.6378, respectively, from feature-pair (aX,aZ). On the other hand, if we take (aZ,vY0) as a major factor, then triplet (aX,aZ,vY0) is a union of two major factors: aX and (aZ,vY0). The SCE drop of this triplet from (aZ,vY0) is calculated as 1.8724, which is very close to the CE drop 1.7232 of aX. That is, these two triplets do not meet the [C2:irreplaceable] criterion, so these two triplets are not order-3 major factors.
  The largest CE drop among all feature-triplets is achieved by (aY,vZ0,pitN). Its successive CE drop from (aY,pitN) is 1.0297(=3.0768−2.0471), which is very significant compared to the CE drop of vZ0. However, if we take both (aY,pitN) and (vZ0,pitN) as two order-2 major factors, then triplet (aY,vZ0,pitN) is a union of (aY,pitN) and (vZ0,pitN). The CE drop of (aY,vZ0,pitN) is calculated as 2.6697, which is less than the sum of the two pairs’ individual CE drops: 3.2402(=1.6399+1.6003). For the same arguments, the following four feature-triplets: (aY,pitN,spinR), (vZ0,pitN,spinR), (aY,pitN,sartSp), and (vZ0,pitN,startSp), are not potential order-3 major factors. These five triplets do not pass meet the criterion [C1:confirmable]; see Figure 5B.
**4:**  If we consider the four-feature-setting, all top 12 quartets on CEs involve pitN, while all 12 quartets of top CE drops do not involve pitN, but 10 out of 12 involve spinR. The 12 quartets on the top CE list all fail to meet the [C2:irreplaceable] criterion, primarily because they contain multiple major factors: either of the two order-1 or three order-2 or four order-3 major factors if we replace features with their highly associated ones, such as startSp for vY0 and spinD for aX, etc. All quartets on the top list of CE drops also fail to meet the criterion [C1:confirmable] by applying Algorithm 2.
**5:**  Five-feature and greater feature settings share the same characteristics with feature-sets in the four-feature setting. They all fail to meet either criterion [C1:confirmable] via Algorithm 2 or criterion [C2: irreplaceable] for the same reasons.

For the response variable $Y=(pfx_{X},pfx_{Z})$, we can confidently identify the following three collections of major factors: {aX,(aZ,vY0)}, {aZ,(vY0,aY)}, and {aZ,(vY0,z0)}. We clearly see the effect of heterogeneity, through pitN is overshadowed by the finite sample phenomenon in the three-feature setting within this MSP of 199 fastball pitchers. No order-3 or higher order major factors are confirmed. Again, the joint effects aX and aZ still overshadow the Magnus effect and the effects of heterogeneity. Therefore, we deepen our investigation with the response variable Y=(aX,aZ).

Likewise we report top ranked CEs and SCE drops in two tables, Table 9 and Table 10, respectively: that is, our results of feature selection based on CEs of all possible feature-sets among the 10 covariate features.

**1:**  In the one-feature setting, spinD and pitN achieve top two ranked CE values. It is not surprising that spinD plays the sole role of order-1 major factor in the identified collections of major factors. It is surprising that, as seen below, we need to bring in aY and vZ0, two separate order-1 major factors, for the sake of improving the CE performances beyond spinD, to construct collections of major factors.
**2:**  In the two-feature setting, the top nine feature pairs achieving the lowest CEs involve spinD. However, none of these nine pairs fulfill the criterion [C2: irreplaceable]. On the other hand, the feature-pair (pitN,aY) and (pitN,vZ0) ranked second and third on the list of top 10 SCE drops, both fail to meet the criterion [C2: irreplaceable], while (pitN,spinR) satisfies both criteria. We also report no candidates for order-2 major factors that do not include pitN. This is a rather surprising observation.
  However, collections {vZ0,(spinR,pitN)} and {aY,(spinR,pitN)} fail to satisfy the criterion [C1: confirmable]. As such, these collections evidently reflect the effects of heterogeneity being overshadowed by the finite sample phenomenon.
**3:**  Considering the three-feature setting, the top 10 feature triplets on CEs are primarily extended from feature pair (spinD,pitN). This is not surprising due to the dominant effect of spinD. They satisfy the criterion [C1: confirmable] (see panel (C) of Figure 5) but not [C2: irreplaceable]. In contrast, the top 10 feature triplets on SCE drops are all involved pitN. They satisfy the criterion [C2: irreplaceable], but not [C1: confirmable].
**4:**  As for the four-feature and greater feature settings, they all fail to meet the criterion [C1:confirmable] based on Algorithm 2. The same example as in sliders, (vZ0,spinR,pitN,aY), has its CE of 0.9875 far beyond the range of the simulated CE distribution of (vZ0,spinR,pitN,ξ), with mean 0.7949 and SD $6.4739\times 10^{-4}$. Furthermore, (vY0,vZ0,spinR,pitN,aY) has its CE of 0.3918 far beyond the range of the simulated CE distribution of (vY0,vZ0,spinR,pitN,ξ), with mean 0.2619 and SD $3.7392\times 10^{-4}$.

The identified three collections of major factors of Y=(aX,aZ) are then (1) {spinD,aY}, (2) {spinD,vZ0}, and (3) {spinD}. We cannot find any collections of major factors consisting of three features.

### 5.3. Comparisons of Summarized Results from Slider and Fastball

We collect and compare major identified factors of various orders via our feature selection protocol on both pitch types, sliders and fastballs, with respect to two different kinds of response variables, Y: $\left(pfx_{X},pfx_{Z}\right)$ and (aX,aZ), in Table 11. Such comparisons reveal fundamental differences of these two pitching dynamics, and at the same time, shed light on the effects of heterogeneity within both MSPs.

When considering $Y=(pfx_{X},pfx_{Z})$ as the response variable, both pitch types select three collections of triplets in an identical format: one order-1 major factor, either aX or aZ, and one biomechanical order-2 major factor. Newton’s second law of force dictates that aX and aZ as directional forces govern the two directional horizontal and vertical movements: $Y=(pfx_{X},pfx_{Z})$. In both pitch types, we also see the heterogeneity effects via pitN commonly interacting with biomechanical features in a format of candidates of order-2 major factors such as, (aY,pitN), (z0,pitN), (spinR,pitN), and (vZ0,pitN). From a biomechanical perspective, we find that slight differences rest on the candidates for order-2 major factors: (spinR,vY0) for sliders, and (aZ,vY0) and (pitN,vY0) for fastballs.

In other words, their differences are primarily tied to “speed” (vY0 or startSp). The coupling effect of (spinR,vY0) might be closely related to the capacity of control of slider pitches. As for fastballs, the speed (vY0 or startSp) is a basic requirement. It is surprising to see that the coupling effects of speed vY0 and vertical acceleration aZ on $Y=(pfx_{X},pfx_{Z})$ are somehow critical in this MSP of fastball pitching dynamics. The candidacy of order-2 major factor (pitN,vY0) indicates that there are significant differences among the 199 fastball pitchers, while this is not the case in sliders.

When Y=(aX,aZ) is the response variable in both pitch types, we found three triplet-based collections of major factors in the sliders, but only two pair-based and one singleton-based collections of major factors in the fastballs. The feature spinD is apparently an order-1 major factor for Y=(aX,aZ) in both pitch types.This clearly points to the fact that the Magnus effect is primarily contained in horizontal and vertical accelerations (aX,aZ).

The effects of heterogeneity among 62 slider pitchers and 199 fastball pitchers are manifested through the same four candidates of order-2 major factors: pitN coupled with one biomechanical member of feature-set {aY,vZ0,spinR,z0}. The impacts of such heterogeneity effects via pitN seem much more severe in fastballs than in sliders.

Further, the presences of (spinR,vX0), (spinR,vY0) as candidates for order-2 major factors in sliders, but not in fastballs, imply characteristic differences between these two pitch types. These two pairs clearly reveal the effects of spinR by coupling the two speed features vX0 and vY0, which are commonly shared by all 62 slider pitchers. Therefore, spinR is most likely linked to better control and reliable stability of slider pitches. In contrast, spinR only plays a minor role in fastballs through one aspect of interacting relations with pitN.

Here, we reiterate that pitN appears in candidates of order-2 major factors by coupling wirh primary biomechanical features under $Y=(pfx_{X},pfx_{Z})$ and Y=(aX,aZ). This evidence strongly indicates that the heterogeneity in these two MSPs must be accommodated before performing any inferences on $Y=(pfx_{X},pfx_{Z})$. This is one of the chief characteristics of MSPs.

### 5.4. Taking Care of Heterogeneity

From the results reported above, it is essential to recognize that pitN neither ever stands alone as an order-1, nor is a member of identified order-2 major factors. Its effects are always revealed through interacting relations with biomechanical and physical features due to it being a candidates for an order-2 major factor. Such interacting relations severely constrain our understanding of the pitching dynamics of both pitch types. In fact, such interacting relations reflect pitN’s diverse associations with a majority of the biomechanical and physical features. As such, these relations are pieces of information on heterogeneity revealed in many feature-specific aspects. What about global information on heterogeneity regarding similarity and dissimilarity among pitchers in both MSPs?

With such global information in mind, it becomes necessary to correspondingly partition the whole collection of pitchers into homogeneous pitcher-groups. By doing so, we want to discover more diverse aspects of pitching dynamics, and at the same time bring out the global distinctions across various homogeneous groups.

**Figure 6:**
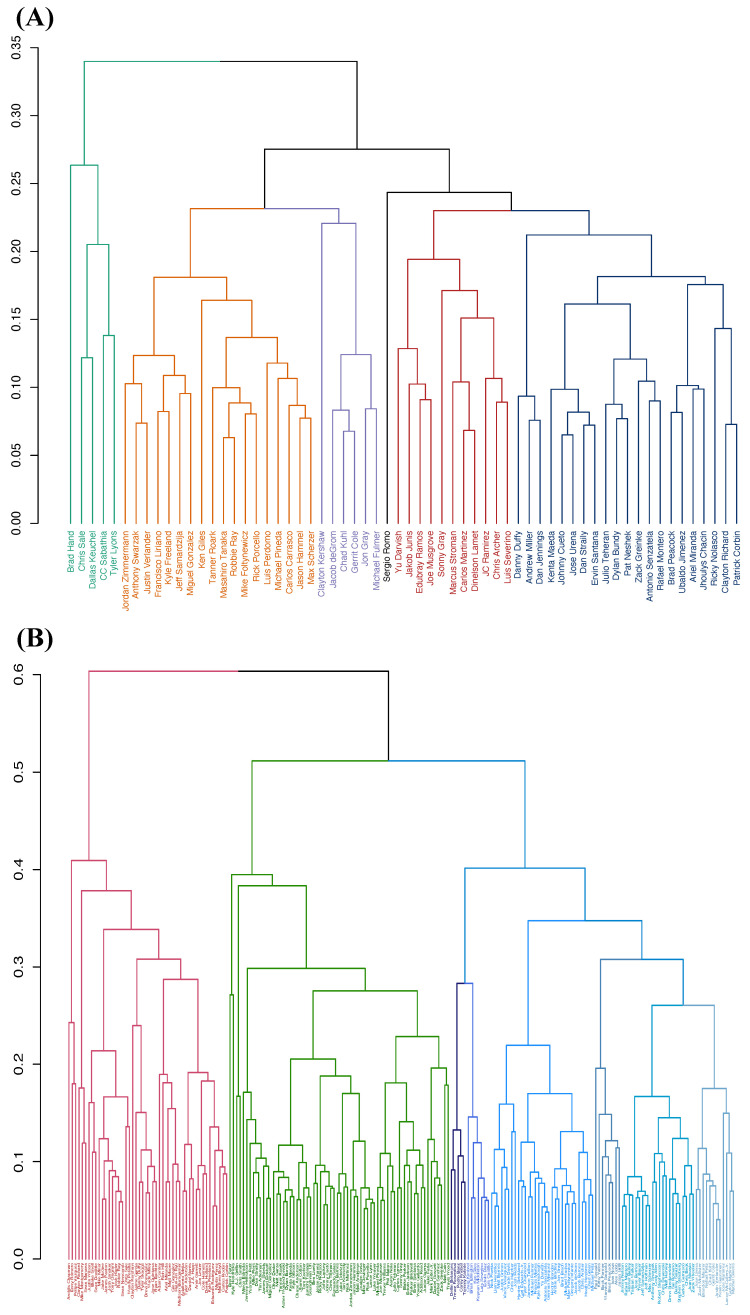
Hierarchical clustering trees marked with pitcher groups: (**A**) 62 slider pitchers; (**B**) 199 fastball pitchers with 3 colored major branches A, B, and C (from left to right). The C-branch is further partitioned into six colored sub-branches: C1 to C6 (left to right).

For the two MSPs of slider pitchers and fastball pitchers, we propose here to perform hierarchical clustering (HC) on the two collections of pitchers with respect to contingency tables of triplet (aX,aZ,vY0) against pitN. These three features in the triplet involve order-1 and order-2 major factors of $Y=(pfx_{X},pfx_{Z})$. The two resulting HC-trees are given in Figure 6. Each HC-tree is marked with various scales of composition of pitcher-groups.

For the sliders, we first consider partitioning the 62 pitchers into five branch-based groups indexed A to E. We designate each branch-based group as one MSP and check whether it embraces homogeneity or not. For such a goal in each branch-based MSP, we conduct feature selection for major factors of the response variable Y=(aX,aZ). We summarize our findings of major factors of these five MSPs in Table 12.

In this table, when checking the criterion [C2: irreplaceable], we make sure that the SCE drop is at least three times that of Condition (a) for any candidate feature-sets. To test for the criterion [C1:confirmable], we use Algorithm 2. The selected collections across these ive MSPs share spinD as the chief order-1 major factor. Then spinD is coupled with various biomechanical features as order-1 major factors with relatively minor effects.

We see that pitN appears as one member of the candidates of order-2 major factors of Y=(aX,aZ) in MSPs defined by branches B, D and E, but not by branches A and C. That is, the branch A- and C- based MSPs embrace homogeneity among pitcher-systems, while branch B-, D-, and E-based MSPs still embrace heterogeneity to a much less extent because the number of such candidates is just two. It is also noted that throughout these five MSPs, pitN achieves the top 2 ranked individual CE drops, which are significantly smaller than the CE drops of the order-1 major factor spinD. We also observe that the SCE drop of (spinD,pitN) is rather small. Therefore, pitN is ruled out as a candidate for an order-1 major factor.

Another essential visible pattern in Table 12 is that the candidates for order-2 major factors of Y=(aX,aZ) become abundant and diverse by involving only biomechanical features in these 5 MSPs. This phenomenon is different from the interacting relations between pitN and biomechanical features in the original MSP consisting of 62 slider pitchers. This fact reflects P.W. Anderson’s “More is Different” perspective of MSPs; its homogeneous components likely reveal more diverse and detailed group-idiosyncratic characteristics.

For fastballs, we first partition the 199 pitchers into three large groups with respect to the three major branches marked and indexed by A, B, and C, in panel (B) of Figure 6. These three large branches are unlikely to embrace homogeneity. Hence, we further partition the C branch into six sub branches marked and indexed by {C1, C2, …, C6}. These six sub branch-based MSPs seemingly embrace homogeneity, as seen in Table 13. That is, all their major factors of Y=(aX,aZ) in the six MSPs do not explicitly contain pitN. Throughout these six MSPs, pitN achieves an individual CE drop that is significantly smaller than the CE drop of spinD.

The MSP C1 does not support any triplets because of the criterion [C1:confirmable]. Only two collections of order-1 major factors are identified within this MSP. Three collections consist of spinD coupling with one of the members of {vZ0,aY,vX0}. The fourth collection is {spinD,spinR}.

The MSP C2 does support one collection of triplets in the format of one order-1 major factor and one order-2 major factor. The other two collections consist of spinD coupled with another less effective order-1 major factor such as vZ0 and aY. There are 13 candidates for order-2 major factors identified, but not a single pair contains pitN as a member. Likewise, in MSP C3, there are two collections of triplets: one order-1 major factor and one order-2 major factor. The alternative is a collection of two order-1 major factors: {spinD,aY}. There are also 12 candidates of order-2 major factors identified without pitN as a member. In a similar fashion, in MSP C5 there are three collections of triplets supported by the data and there are 13 candidates for order-2 that do not include pitN. In MSP C6, identified collections are similar to those found in MSP C3.

In MSP C4, the first two selected collections of triplets share an unusual format: two order-2 major factors. The members of these two triplets are all biomechanical features without spinD. The third collection is {spinD,aY}. There are no other candidates for order-2 major factors.

Overall, we identify abundant candidates for order-2 major factors that do not involve pitN at all across the six MSPs. In other words, the effect of heterogeneity seems to have disappeared.

Up to this point, our slider and fastball examples strongly suggest that a rigorous way for analyzing any MSPs containing a large number of complex systems must take the following steps. Firstly, we explore and understand how the complexity of the underlying dynamics interacts with heterogeneity. Secondly, this exploration leads us to find a proper clustering and grouping scheme, such that similar members of the original collection of systems are grouped together in order to embrace homogeneity. Thirdly, when such homogeneity is achieved across all subdivided groups, a more diverse and finer scale group-specific complexity can be discovered. This is because a group of similar systems will collectively retain a much larger number of data points than any of its single member systems. This approach for analyzing any large MSPs by going from the heterogeneity of the whole to the diverse homogeneity of its constituents is a way of seeing and understanding “More is Different” in MSPs.

## 6. Self-Esteem across Gender and Age Human Complex Systems

In the very last section of this paper, we explore a potential merit of MSP study in a research area where psychology, psychiatry, and sociology intersect. The scientific question can be simply stated as: do people of different genders and ages respond to the Rosenberg Self-Esteem Scale differently? Prof. Morris Rosenberg studied dynamic changes of late adolescents’ self-image in his well known 1965 book [10]. Boys and girls of 15 to 18 years of age, the so-called late adolescence, must make drastic self-image changes in response to drastic physiological and psychological developments due to changes in their own bodies, as well as the different societal potentials being discovered and the many career decisions that must be made. In order to study this topic, he designed the popular Rosenberg Self-Esteem Scale with 10 questions for his study subjects.

More than half of a century has passed since the publication of his book. Intuitively speaking, human self-image might be shaped, for example, by the abundant information available on the Internet. In fact, nowadays, all age and gender groups are exposed to and share a huge amount of information in regard to our world’s drastic environmental and technological changes. Do such visible world-wide changes in our living conditions motivate and affect people’s self-image differently or similarly?

Nowadays, the Rosenberg Self-Esteem Scale has become a popular on-line test. Many persons who are far outside the original domain of application of this test have taken the test and their results are recorded. A Rosenberg Self-Esteem Scale data set from Kaggle can be found via the following link: https://www.kaggle.com/yamqwe/rosenberg-selfesteem-scale (accessed on 3 January 2022). When addressing such data, it is reasonable to first ask whether seemingly diverse systems defined across gender and age axes are equal through the perspective of the Rosenberg Self-Esteem Scale, This is one version of an MSP without specifically targeted Re–Co dynamics. Only by arriving at an answer to this MSP question can the second question be formulated accordingly.

Under the MSP setting, the first question can be stated as: does potential heterogeneity exist across spans of the age-axis and gender-axis or not? This is a rather interesting issue from the perspective of psychiatry, psychology, and sociology. We want to shed some light on this issue via the computational major factor selection method developed and illustrated in the previous sections.

This data set consists of the results of 47,974 subjects who took the online Rosenberg Self-Esteem Scale. We deleted subjects younger than 10 or older than 70. Subjects who did not indicate their gender information are collected into the “none was chosen” category of the gender variable. There are 10 questions in the Rosenberg Self-Esteem Test. Each subject can respond on the following scale: 1= strongly disagree, 2= disagree, 3= agree, and 4= strongly agree (0= no answer).

$Q_{1}$. I feel that I am a person of worth, at least on an equal plane with others.
$Q_{2}$. I feel that I have a number of good qualities.
$Q_{3}$. All in all, I am inclined to feel that I am a failure.
$Q_{4}$. I am able to do things as well as most other people.
$Q_{5}$. I feel I do not have much to be proud of.
$Q_{6}$. I take a positive attitude toward myself.
$Q_{7}$. On the whole, I am satisfied with myself.
$Q_{8}$. I wish I could have more respect for myself.
$Q_{9}$. I certainly feel useless at times.
$Q_{10}$. At times I think I am no good at all.

Here we propose one fundamental way of resolving the aforementioned issue by looking into all possible response-to-covariate (Re–Co) dynamics and checking the effects of heterogeneity. For expositional simplicity, we use only one instance as an example. Given $Q_{1}$, it is taken as a 1D response feature $Y=Q_{1}$, while the rest of the nine questions $Q_{2}$–$Q_{10}$ are taken as covariate features. These 10 features are ordinal. We also take Age and Gender as two additional covariate features. Specifically, Age takes categorical-membership values among the ordinal age-groups [10,14], [15,18], [19,22], [23,30], [31,40], [41,50], [51,60], and [61,70], while $V_{11}=Gender$ takes categorical values of 1=“male”, 2=“female”, 3= “other”; 0=”none was chosen”. That is, the MSP under study here is specified by the collection of complex systems encoded with the bivariate categories of (Age,Gender).

**Figure 7:**
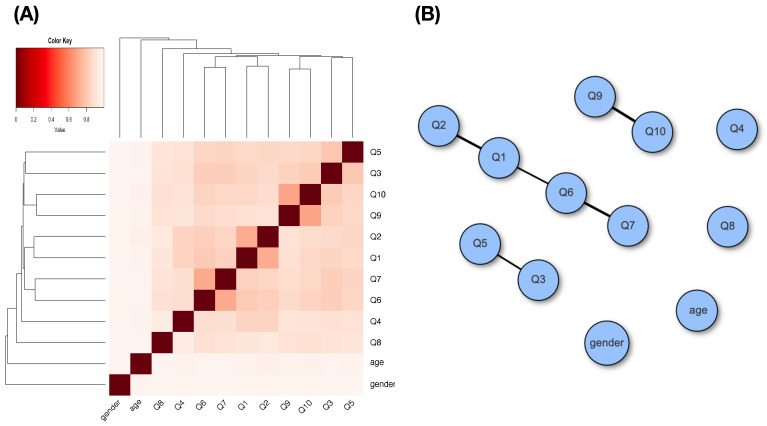
Associative patterns among 12 features of Rosenberg Self-Esteem Scale:(**A**) heatmap based on MCE-matrix; (**B**) network built with linkages with thickness proportional to one minus pairwise MCE and subject to a threshold 0.2 (=1.0−MCE).

The pairwise associations among these 12 categorical features are presented through an MCE-based heatmap and its network in Figure 7. A glimpse of an associative map of these 12 features is seen through the three small communities in the network. When showing heterogeneity effects via our major factor selection, Age and Gender will be treated as two separate covariate features, as is demonstrated in Table 14 and Table 15.

Selection of major factors in the Self-esteem test example with heterogeneity.

**1**  In the one-feature setting of Table 14 and Table 15, $Q_{2}$ achieves the smallest CE with the largest CE drop: 0.3364. $Q_{6}$ achieves the second ranked CE with a CE drop of 0.2443. These two features are potential order-1 major factors. $Q_{7}$, $Q_{4}$ and $Q_{3}$ are ranked third, fourth, and fifth, with CE drops near 0.2000. In sharp contrast, Age and Gender individually have rather low CE drops. These two pieces of evidence indicate the absence of heterogeneity effects. This fact is significantly distinct from the presence of effects of heterogeneity discussed in previous sections for our designed MSP and all MLB-based MSP examples.
**2**  In the two-feature setting, we can see that the SCEs are all significantly lower than corresponding CE drops among features from $Q_{3}$ to $Q_{10}$. That is, all feature-pairs fail the criterion [C2: unreplacable], including pairs involving Age and Gender. Therefore, no order-2 major factors can be seen. The primary reason for this phenomenon is that $Q_{2}$ is associated with all other covariate features to the degree that there are no pairs satisfying the ecological effects.

We conclude that $Q_{2}$ is the solo order-1 major factor of the $Q_{1}$-based Re–Co dynamics and that the effects of heterogeneity of Age and Gender are absent. In other words, from the perspective of the $Q_{1}$-based Re–Co dynamics, all age-vs-gender defined systems are not heterogeneous. To fully resolve this issue, we need all possible Re–Co dynamics. This task will be undertaken in a separate report.

**Figure 8:**
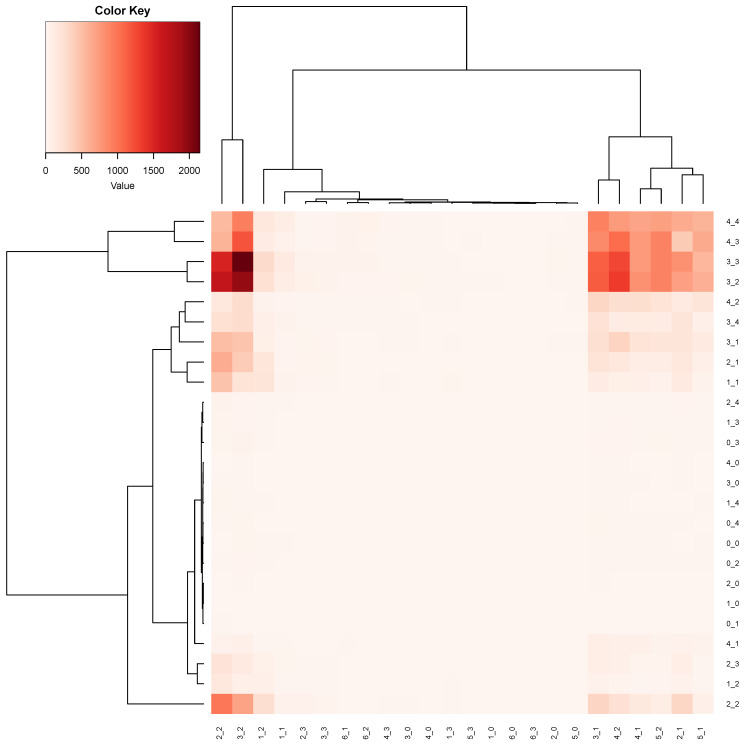
Decomposing the collection of systems defined by Age and Gender into homogeneous groups in the Rosenberg Self-Esteem Scale. Bivariate-encoded Age-vs-Gender systems are arranged along the column-axis, while the bivariate-encoded Q2-vs-Q6 categories are arranged along the row-axis. An HC-tree is superimposed on the column-axis for identifying homogeneous groups of systems.

To further address this issue in depth, we go into the phase of decomposing the collection of complex systems into homogeneous groups; that is, the categories of the bivariate (Age,Gender) are clustered into homogeneous groups, as shown in Figure 8. Then, we apply our major factor selection protocol to further check whether the effects of Age or Gender exist within any of these homogeneous groups of systems.

Based on Figure 8, we select three obvious cluster-based groups: $R_{1}=\left\{\left(2,2\right),\left(3,2\right)\right\}$, $R_{2}=\left\{\left(4,2\right),\left(3,1\right)\right\}$, and $R_{3}=\left\{\left(2,1\right),\left(4,1\right),\left(5,1\right),\left(5,2\right)\right\}$. The sample size is 16,800 for $R_{1}$, 11,721 for $R_{2}$, and 15,312 for $R_{3}$. The single major factor collection $\left\{Q_{2}\right\}$ is selected for $R_{3}$ and $R_{3}$, while a collection of two order-1 major factors $\left\{Q_{2},Age\right\}$ is selected for $R_{2}$, since $Q_{2}$ and Age jointly achieve a SCE drop of (0.0046) against the CE drop of Age of (0.0024). Thus, the ecological effect is satisfied. In addition, Age also meets the criterion [C1:confirmable], since its CE of 1.1713 is below the CE distribution of ε (having the same number of categories of Age) with a mean of 1.1736 and SD of $1.2193\times 10^{-4}$. Therefore, $Q_{2}$ and Age can both be order-1 major factors. Nevertheless, $Q_{2}$ is still the dominant order-1 major factor. Hence, we still conclude with a coherent result: systems within these three homogeneous groups are not heterogeneous from the perspective of $Q_{1}$ dynamics.

At the end of this example, we remark that the widely used Rosenberg Self-Esteem Scale was conceptualized by its author as a single-factor scale with scores ranging along a continuum of low self-esteem to high self-esteem [10]. From this perspective, our identification of $Q_{2}$ as the single major factor is seemingly coherent. Further, when three versions of rewording on Rosenberg Self-Esteem Scale were devised and a much smaller data set was collected and analyzed [18], the results of factor analysis [19] indeed indicated that the original version fits a two-factor model, while positively and negativey reworded versions fit single-factor models. It is noted that these results based on factor analysis are fundamentally irrelevant to the issue under study here.

## 7. Conclusions

We demonstrated the versatile merits of a CEDA-based protocol for selecting major factors of various orders as a proper way of studying any MSP. First, it is essential to reveal patter -information of “complexity interacting with heterogeneity” embedded within MSPs. Secondly, the heterogeneity embedded within the whole ensemble of complex systems needs to be broken down into homogeneity embraced by constituent groups of similar complex systems. Finally, similarity-based homogeneity and largeness of the data set will enable each constituent group to manifest detailed multiscale characteristics via major factors of various orders. This is taken as the true implication of P. W. Anderson’s “More is Different” under a structured data setting. This MSP implication of breaking heterogeneity for the sake of unraveling possibly distinct collections of hidden major factors across homogeneous parts may be fundamental and critical in this Big Data era.

With an understanding of such an MSP implication, we further demonstrate its merits in formulating resolutions to a scientific problem related to the Rosenberg Self-Esteem Scale in psychology and sociology. This demonstration helps us recognize the degree of the spread of MSPs in the real world and simultaneously indicates the huge potential of our computational major factor selection techniques.

As scientists, we want to prevent data-driven understandings being twisted and information content being compromised. Nowadays, MSPs can be ubiquitously found in diverse research fields and real-world businesses. Proper discovery of data’s authentic information content and true understanding of it should greatly help our societies. In this paper, we demonstrate that data-driven understandings can be supported by visible and explainable relational patterns found on the simple platform of contingency tables. This is essential and important for data analysis in this Big Data era.

Furthermore, we emphasize that our CEDA computations work for all data types. This is an essential virtue of data analysis, since its categorical nature involves features of any data types. By employing such a categorical nature in data, the contingency table platform becomes natural, as do information theoretical measurements. Consequently, the pattern-formation brought out by conditional entropy and mutual information is natural, so it reveals and facilitates understanding pertaining to MSPs of interest. In other words, this CEDA-based data analysis is an effective way of studying an ensemble of complex systems.

By visualizing how complexity interacts with heterogeneity and how homogeneity unravels many more major factors in the two MLB-related MSPs, we recognize the fact that a group of similar pitchers’ collective pitching dynamics is indeed much more involved in many aspects than the whole collection of pitchers, as well as a single pitcher’s pitching dynamics. This recognition allows us to fundamentally resolve the issue of how to properly compare the pitching dynamics of a group of pitchers.

By resolving these real-world MSPs, we also demonstrated how to successfully implement the two criteria [C1: confirmable] and [C2: irreplaceable] together with reliability checks. By recognizing existential heterogeneity and exploring its complicated effects and then teasing out diverse versions of homogeneity in all constituent groups, our various collections of major factors bring out the common governing mechanisms shared by all involved complex systems and, at the same time, they manifest distinctive mechanisms that characterize each of the identified groups of similar complex systems. The reliability check using Algorithm 2 is critical when subject to the finite sample phenomenon, even in the Big Data era.

Finally, we summarize the chief concept underlying our CEDA-based feature selection for major factors: “Let data’s categorical nature assemble freely and naturally to shed light on complexity, heterogeneity, and homogeneity in MSPs”.

## Figures and Tables

**Table 1:** Top 12 ranked successive CE drops of feature-sets across one-feature to three-feature settings of the designed MSP. CE of *Y* is equal to 2.4338.

One-Feature	SCE Drop	Two-Feature	SCE Drop	Three-Feature	SCE Drop
$V_{2}$	0.3332	$V_{1}\_V_{4}$	0.1350	$V_{1}\_V_{4}\_V_{5}$	1.0734
$V_{1}$	0.0106	$V_{1}\_V_{5}$	0.1349	$V_{1}\_V_{3}\_V_{6}$	1.0708
$V_{11}$	0.0017	$V_{1}\_V_{3}$	0.1336	$V_{1}\_V_{3}\_V_{4}$	1.0706
$V_{5}$	0.0016	$V_{1}\_V_{6}$	0.1322	$V_{1}\_V_{3}\_V_{5}$	1.0706
$V_{10}$	0.0015	$V_{1}\_V_{8}$	0.1229	$V_{1}\_V_{4}\_V_{6}$	1.0682
$V_{3}$	0.0014	$V_{1}\_V_{12}$	0.1214	$V_{1}\_V_{5}\_V_{6}$	1.0682
$V_{8}$	0.0014	$V_{1}\_V_{9}$	0.1213	$V_{1}\_V_{3}\_V_{9}$	1.0327
$V_{4}$	0.0013	$V_{1}\_V_{10}$	0.1207	$V_{1}\_V_{3}\_V_{12}$	1.0326
$V_{9}$	0.0013	$V_{1}\_V_{11}$	0.1201	$V_{1}\_V_{4}\_V_{12}$	1.0308
$V_{6}$	0.0013	$V_{1}\_V_{7}$	0.1193	$V_{1}\_V_{5}\_V_{12}$	1.0300
$V_{12}$	0.0012	$V_{1}\_V_{2}$	0.1035	$V_{1}\_V_{3}\_V_{10}$	1.0300
$V_{7}$	0.0011	$V_{10}\_V_{11}$	0.0194	$V_{1}\_V_{5}\_V_{9}$	1.0289

**Table 2:** Designed MSP: comparisons of collections of major factors based on the whole data set with heterogeneity and homogeneous groups.

Whole or Group	Order-1 MF	Order-3 MF	Alternative MF
Whole-data	$V_{2}$	none confirmed	none confirmed
$G_{1}$	$V_{2}$	$\left(V_{4},V_{5},V_{6}\right)$	none confirmed
$G_{2}$	$V_{2}$	$\left(V_{3},V_{5},V_{6}\right)$	none confirmed
$G_{3}$	$V_{2}$	$\left(V_{3},V_{4},V_{6}\right)$	none confirmed
$G_{4}$	$V_{2}$	$\left(V_{3},V_{4},V_{5}\right)$	none confirmed
$G_{5}$	$V_{2}$	none confirmed	none confirmed

**Table 3:** Sliders: 12 top ranked CEs of five settings of feature-sets for response variable $Y=(pfx_{X},pfx_{Z})$. CE of Y is 4.6890.

One-Feature	CE	Two-Feature	CE	Three-Feature	CE	Four-Feature	CE	Five-Feature	CE
aX	2.7631	aZ_aX	0.9264	aZ_aX_pitN	0.6617	aY_aZ_aX_pitN	0.3694	aY_aZ_vZ0_aX_pitN	0.1070
aZ	2.8121	aX_spinD	1.8411	aZ_vY0_aX	0.6922	aZ_vZ0_aX_pitN	0.3726	aY_aZ_vZ0_z0_aX	0.1313
spinD	3.0063	aZ_spinD	1.8678	aZ_aX_startSp	0.6946	aZ_vY0_aX_pitN	0.4377	aY_aZ_vY0_vZ0_aX	0.1400
pitN	3.5526	aZ_pitN	1.9508	aZ_aX_spinR	0.8216	aZ_aX_startSp_pitN	0.4390	aY_aZ_vZ0_aX_startSp	0.1430
x0	4.3582	aX_pitN	2.1953	aZ_z0_aX	0.8238	aZ_aX_spinR_pitN	0.4537	aY_aZ_vY0_z0_aX	0.1439
vX0	4.3719	spinD_pitN	2.4467	aY_aZ_aX	0.8251	aZ_vY0_vZ0_aX	0.4596	aY_aZ_z0_aX_startSp	0.1448
startSp	4.4369	aZ_vX0	2.4929	aZ_vZ0_aX	0.8342	aZ_vY0_z0_aX	0.4603	aZ_vY0_vZ0_z0_aX	0.1479
vY0	4.4392	aZ_x0	2.4961	aZ_vX0_aX	0.8388	aZ_z0_aX_startSp	0.4618	aZ_vZ0_z0_aX_startSp	0.1485
vZ0	4.5399	aZ_vY0	2.5263	aZ_x0_aX	0.8505	aZ_vZ0_aX_startSp	0.4627	aY_aZ_vZ0_aX_spinR	0.1538
spinR	4.5482	aZ_startSp	2.5290	aZ_aX_spinD	0.8845	aY_aZ_vY0_aX	0.4635	aY_aZ_z0_aX_spinR	0.1556
z0	4.6012	aX_startSp	2.5462	aZ_spinD_pitN	1.3791	aZ_vX0_aX_pitN	0.4640	aY_aZ_vY0_aX_pitN	0.1575
aY	4.6261	vY0_aX	2.5463	aY_aZ_pitN	1.4649	aY_aZ_aX_startSp	0.4663	aY_aZ_aX_startSp_pitN	0.1594

**Table 4:** Sliders: 12 top ranked CE drops of four settings of feature-sets for the response variable $Y=(pfx_{X},pfx_{Z})$.

One-Feature	SCE Drop	Two-Feature	SCE Drop	Three-Feature	SCE Drop	Four-Feature	SCE Drop
aX	1.9261	aZ_aX	1.8367	aY_vZ0_pitN	1.1533	aY_vY0_vZ0_z0	1.6601
aZ	1.8771	aZ_spinD	0.9443	aY_vZ0_z0	1.0929	aY_vZ0_z0_spinR	1.6535
spinD	1.6829	aX_spinD	0.9220	vZ0_z0_spinR	1.0343	aY_vZ0_z0_startSp	1.6521
pitN	1.1366	aZ_pitN	0.8614	aY_z0_spinR	1.0241	aY_vX0_vZ0_z0	1.6374
x0	0.3310	aX_pitN	0.5679	aY_vZ0_spinR	1.0032	aY_vX0_vZ0_spinR	1.6105
vX0	0.3173	spinD_pitN	0.5595	vY0_vZ0_z0	0.9929	aY_vY0_vZ0_spinR	1.5936
startSp	0.2523	vZ0_pitN	0.4544	vZ0_z0_startSp	0.9863	aY_vZ0_spinR_startSp	1.5859
vY0	0.2500	aY_pitN	0.4456	aY_vY0_z0	0.9850	aY_vY0_z0_spinR	1.5651
vZ0	0.1493	spinR_startSp	0.3788	aY_vX0_z0	0.9798	aY_vX0_vY0_z0	1.5645
spinR	0.1410	vY0_spinR	0.3772	aY_z0_startSp	0.9784	aY_vX0_z0_spinR	1.5644
z0	0.0880	vX0_startSp	0.3728	aY_vY0_vZ0	0.9674	vY0_vZ0_z0_spinR	1.5629
aY	0.0631	vX0_vY0	0.3722	aY_vZ0_startSp	0.9589	aY_vX0_vY0_vZ0	1.5616

**Table 5:** Sliders: 10 top ranked CEs of five settings of feature-sets for the response variable Y=(aX,aZ). CE of Y is 4.7213.

One-Feature	CE	Two-Feature	CE	Three-Feature	CE	Four-Feature	CE	Five-Feature	CE
spinD	3.0301	spinD_pitN	2.4891	aY_vZ0_pitN	1.9577	aY_vZ0_spinR_pitN	0.9624	aY_vY0_vZ0_spinR_pitN	0.3837
pitN	3.6086	vX0_spinD	2.8168	vZ0_spinD_pitN	1.9577	aY_vZ0_spinD_pitN	0.9732	aY_vZ0_spinR_startSp_pitN	0.3873
x0	4.3981	x0_spinD	2.8689	aY_spinD_pitN	1.9657	aY_vY0_vZ0_pitN	0.9867	aY_vX0_vZ0_spinR_pitN	0.3922
vX0	4.4110	vZ0_spinD	2.8779	vX0_spinD_pitN	2.1228	aY_vZ0_startSp_pitN	0.9961	aY_vX0_vZ0_z0_spinR	0.4034
startSp	4.4923	spinD_startSp	2.8848	spinD_startSp_pitN	2.1355	aY_vX0_vZ0_pitN	1.0258	aY_vY0_vZ0_z0_spinR	0.4053
vY0	4.4950	vY0_spinD	2.8854	vY0_spinD_pitN	2.1387	aY_vZ0_z0_pitN	1.0853	aY_vZ0_spinR_spinD_pitN	0.4074
vZ0	4.5698	spinR_spinD	2.9170	spinR_spinD_pitN	2.1541	aY_vX0_spinD_pitN	1.2414	aY_vZ0_z0_spinR_startSp	0.4098
spinR	4.5811	z0_spinD	2.9294	z0_spinD_pitN	2.1892	aY_spinR_spinD_pitN	1.2517	aY_vX0_vY0_vZ0_pitN	0.4125
z0	4.6370	aY_spinD	2.9413	aY_spinR_pitN	2.2981	vZ0_spinR_spinD_pitN	1.2582	aY_vX0_vY0_vZ0_z0	0.4153
aY	4.6526	vZ0_pitN	3.1350	vZ0_spinR_pitN	2.3014	vY0_vZ0_spinD_pitN	1.2639	aY_vX0_vZ0_startSpeed_pitN	0.4176

**Table 6:** Sliders: 10 top ranked CE drops of four settings of feature-sets for the response variable Y=(aX,aZ).

One-Feature	SCE Drop	Two-Feature	SCE Drop	Three-Feature	SCE Drop	Four-Feature	SCE Drop
spinD	1.6912	spinD_pitN	0.5410	aY_vZ0_pitN	1.1774	aY_vY0_vZ0_z0	1.6804
pitN	1.1127	vZ0_pitN	0.4736	aY_vZ0_z0	1.1105	aY_vZ0_z0_startSp	1.6727
x0	0.3233	aY_pitN	0.4644	vZ0_z0_spinR	1.0418	aY_vZ0_z0_spinR	1.6705
vX0	0.3104	spinR_startSp	0.3855	aY_z0_spinR	1.0417	aY_vX0_vZ0_z0	1.6459
startSp	0.2290	vY0_spinR	0.3832	aY_vZ0_spinR	1.0220	aY_vX0_vZ0_spinR	1.6207
vY0	0.2263	vX0_startSp	0.3641	vY0_vZ0_z0	1.0173	aY_vY0_vZ0_spinR	1.6133
vZ0	0.1515	vX0_vY0	0.3627	aY_vY0_z0	1.0129	aY_vZ0_spinR_startSp	1.6066
spinR	0.1402	x0_startSp	0.3625	vZ0_z0_startSp	1.0103	aY_vY0_z0_spinR	1.5845
z0	0.0844	vY0_x0	0.3597	aY_z0_startSp	1.0040	vY0_vZ0_z0_spinR	1.5809
aY	0.0687	vX0_spinR	0.3530	aY_vX0_z0	0.9978	aY_vX0_z0_spinR	1.5770

**Table 7:** Fastballs: 12 top ranked CE of five settings of feature-sets for the response variable $Y=(pfx_{X},pfx_{Z})$. The CE of Y is 4.7167.

One-Feature	CE	Two-Feature	CE	Three-Feature	CE	Four-Feature	CE	Five-Feature	CE
spinD	2.9688	aZ_aX	1.7424	aZ_vY0_aX	1.0888	aZ_vY0_aX_pitN	0.7295	aY_aZ_vZ0_spinR_pitN	0.2540
aX	2.9935	aZ_spinD	1.9326	aZ_aX_startSp	1.1046	aZ_vZ0_aX_pitN	0.7318	aY_aZ_vZ0_aX_pitN	0.2638
pitN	3.5048	aZ_pitN	2.0718	aZ_aX_pitN	1.1695	aZ_aX_startSp_pitN	0.7407	aY_aZ_vY0_vZ0_pitN	0.2763
aZ	3.5102	aX_spinD	2.4713	aZ_spinD_pitN	1.2602	aY_aZ_vZ0_pitN	0.7540	aY_aZ_vZ0_startSp_pitN	0.2806
vX0	4.0981	spinD_pitN	2.5521	aZ_vY0_spinD	1.2951	aY_aZ_aX_pitN	0.7687	aZ_vY0_vZ0_aX_pitN	0.2944
x0	4.1266	aX_pitN	2.5772	aZ_spinD_startSp	1.3106	aZ_vZ0_spinD_pitN	0.8125	aZ_vZ0_aX_startSp_pitN	0.2984
aY	4.5772	vX0_spinD	2.8220	aZ_vZ0_pitN	1.5956	aZ_vY0_spinD_pitN	0.8190	aY_aZ_vZ0_spinD_pitN	0.3062
vZ0	4.5960	aY_spinD	2.8287	aZ_vY0_pitN	1.5965	aZ_aX_spinR_pitN	0.8211	aZ_vZ0_aX_spinR_pitN	0.3080
z0	4.6173	aZ_vY0	2.8443	aZ_startSp_pitN	1.6070	aZ_spinD_startSp_pitN	0.8293	aY_aZ_vY0_aX_pitN	0.3249
spinR	4.6676	aY_aX	2.8474	aY_aZ_pitN	1.6196	aY_aZ_spinD_pitN	0.8453	aY_aZ_aX_startSp_pitN	0.3306
startSp	4.6728	vZ0_spinD	2.8520	aZ_aX_spinD	1.6315	aZ_vZ0_spinR_pitN	0.8567	aY_aZ_vX0_vZ0_pitN	0.3307
vY0	4.6740	aZ_startSp	2.8558	aY_aZ_aX	1.6560	aZ_vY0_vZ0_pitN	0.8660	aZ_vY0_vZ0_spinR_pitN	0.3348

**Table 8:** Fastballs: 12 top ranked CE drops of four settings of feature-sets for the response variable $Y=(pfx_{X},pfx_{Z})$.

One-Feature	SCE Drop	Two-Feature	SCE Drop	Three-Feature	SCE Drop	Four-Feature	SCE Drop
spinD	1.7480	aZ_pitN	1.4329	aY_vZ0_pitN	1.0297	vY0_vZ0_z0_spinR	1.4772
aX	1.7232	aZ_aX	1.2511	vZ0_spinR_pitN	0.8179	aY_vZ0_z0_spinR	1.4731
pitN	1.2120	aZ_spinD	1.0362	aY_spinR_pitN	0.7552	vZ0_z0_spinR_startSp	1.4700
aZ	1.2065	aZ_vY0	0.6659	vY0_vZ0_pitN	0.7349	aY_vY0_vZ0_spinR	1.3818
vX0	0.6186	aZ_startS	0.6544	vZ0_startSp_pitN	0.7316	aY_vZ0_spinR_startSp	1.3622
x0	0.5901	aZ_vX0	0.6232	aZ_spinD_pitN	0.6724	aY_vY0_z0_spinR	1.3555
aY	0.1395	aZ_x0	0.5920	aY_vY0_pitN	0.6717	aY_vY0_vZ0_z0	1.3527
vZ0	0.1207	aX_spinD	0.4975	aY_startSp_pitN	0.6612	aY_z0_spinR_startSp	1.3390
z0	0.0995	aY_pitN	0.4280	aZ_vY0_aX	0.6536	aY_vZ0_z0_startSp	1.3321
spinR	0.0491	spinD_pitN	0.4167	aZ_vX0_vY0	0.6467	vX0_vY0_vZ0_spinR	1.1691
startSp	0.0439	aX_pitN	0.4163	aZ_vX0_startSp	0.6465	vY0_vZ0_x0_spinR	1.1612
vY0	0.0428	vZ0_pitN	0.3883	aZ_aX_startSp	0.6378	vX0_vZ0_spinR_startSp	1.1605

**Table 9:** Fastballs: 10 top ranked CE drops of four settings of feature-sets for the response variable Y=(aX,aZ). CE of Y is 4.7000.

One-Feature	CE	Two-Feature	CE	Three-Feature	CE	Four-Feature	CE	Five-Feature	CE
spinD	2.9447	spinD_pitN	2.4991	aY_spinD_pitN	1.9962	aY_vZ0_spinR_pitN	0.9875	aY_vY0_vZ0_spinR_pitN	0.3918
pitN	3.4559	aY_spinD	2.7193	vZ0_spinD_pitN	2.0159	aY_vZ0_spinD_pitN	1.0371	aY_vZ0_spinR_spinD_pitN	0.3965
vX0	4.0743	vX0_spinD	2.7908	aY_vZ0_pitN	2.0286	aY_vY0_vZ0_pitN	1.0750	aY_vZ0_spinR_startSp_pitN	0.3989
x0	4.1036	vZ0_spinD	2.8114	spinR_spinD_pitN	2.1612	aY_vZ0_startSp_pitN	1.0889	aY_vZ0_z0_spinR_pitN	0.4428
aY	4.4743	x0_spinD	2.8391	spinD_startSp_pitN	2.1878	aY_vX0_vZ0_pitN	1.1993	aY_vX0_vZ0_spinR_pitN	0.4434
vZ0	4.5634	spinD_startSp	2.8422	vY0_spinD_pitN	2.1966	aY_vZ0_z0_pitN	1.1993	aY_vY0_vZ0_spinD_pitN	0.4435
startSp	4.5861	vY0_spinD	2.8526	vX0_spinD_pitN	2.2366	vZ0_spinR_spinD_pitN	1.2426	aY_vZ0_spinD_startSp_pitN	0.4504
vY0	4.5964	z0_spinD	2.8873	z0_spinD_pitN	2.2529	aY_spinR_spinD_pitN	1.2773	aY_vX0_vY0_vZ0_pitN	0.4966
z0	4.6030	spinR_spinD	2.8900	vZ0_spinR_pitN	2.2775	vY0_vZ0_spinD_pitN	1.3018	aY_vY0_vZ0_z0_pitN	0.5008
spinR	4.6380	aY_pitN	3.0293	aY_spinR_pitN	2.2950	vZ0_spinD_startSp_pitN	1.3043	aY_vX0_vZ0_startSp_pitN	0.5064

**Table 10:** Fastballs: 10 top ranked CE drops of four settings of feature-sets for the response variable Y=(aX,aZ).

One-Feature	SCE Drop	Two-Feature	SCE Drop	Three-Feature	SCE-Drop	Four-Feature	SCE Drop
spinD	1.7553	spinD_pitN	0.4457	aY_vZ0_pitN	1.0006	vY0_vZ0_z0_spinR	1.4383
pitN	1.2441	aY_pitN	0.4266	vZ0_spinR_pitN	0.8026	vZ0_z0_spinR_startSp	1.4257
vX0	0.6257	vZ0_pitN	0.3757	aY_spinR_pitN	0.7343	aY_vZ0_z0_spinR	1.4129
x0	0.5964	spinR_pitN	0.2689	vY0_vZ0_pitN	0.7276	aY_vY0_vZ0_spinR	1.3119
aY	0.2257	aY_x0	0.2566	vZ0_startSp_pitN	0.7262	aY_vY0_z0_spinR	1.3024
vZ0	0.1366	aY_vX0	0.2550	aY_vY0_pitN	0.6490	aY_vZ0_spinR_startSp	1.2933
startSp	0.1139	startSp_pitN	0.2485	aY_startSp_pitN	0.6422	aY_z0_spinR_startSp	1.2879
vY0	0.1036	vY0_pitN	0.2380	vZ0_z0_pitN	0.6047	aY_vY0_vZ0_z0	1.2832
z0	0.0970	aY_spinD	0.2255	spinR_startSp_pitN	0.5933	aY_vZ0_z0_startSp	1.2641
spinR	0.0620	z0_pitN	0.2069	vX0_vZ0_pitN	0.5911	vX0_vY0_vZ0_spinR	1.1406

**Table 11:** Comparison of major factors of sliders and fastballs with respect to $Y=(pfx_{X},pfx_{Z})$ and Y=(aX,aZ).

Pitch Type	Y	Order-1 MF	Order-2 MF	Alternative MFs	Alternative MFs
slider	$\left(pfx_{X},pfx_{Z}\right)$	aZ	(z0,vY0)	{aX,(z0,vZ0)}	{aX,(aY,vZ0)}
slider	(aX,aZ)	{spinD}	(z0,vZ0)	{spinD,(aY,vZ0)}	{spinD,(aY,z0)}
fastball	$\left(pfx_{X},pfx_{Z}\right)$	aX	(aZ,vY0)	{aZ,(vY0,aY)}	{aZ,(vY0,z0)}
fastball	(aX,aZ)	spinD, aY	none confirmed!	{spinD,vZ0}	{spinD}

**Table 12:** Table of major factors of Y=(aX,aZ) in five groups of slider pitchers (A–E).

Pitch Groups	CE of Y	Order-1 MF	Order-2 MF	Alternative MFs	Alternative MFs
A	3.3574	spinD, aY	None confirmed!	None	None
B	4.2448	spinD, vX0	None confirmed!	{spinD,x0}	{spinD,vZ0}
C	4.1125	spinD, z0	None confirmed!	{spinD,aY}	{spinD,spinR}
D	4.0750	spinD, vZ0	None confirmed!	{spinD,z0}	{spinD,aY}
E	4.6577	spinD, vZ0	None confirmed!	{spinD,aY}	{spinD}

**Table 13:** Table of major factors of Y=(aX,aZ) in six subgroups of the C group of pitchers.

Pitch Groups	CE of Y	Order-1 MF	Order-2 MF	Alternative MFs	Alternative MFs
C1	3.6074	spinD,vZ0	none confirmed	{spinD,aY}	{spinD,vX0}
C2	3.8715	spinD	(aY,vZ0)	{spinD,vZ0}	{spinD,aY}
C3	4.1491	spinD	(aY,vZ0)	{spinD,(aY,spinR)}	{spinD,aY}
C4	3.5763	None confirmed!	(vZ0,spinR), (aY,vZ0)	{(aY,vZ0),(vZ0,z0)}	{spinD,aY}
C5	3.9358	spinD	(aY,z0)	{spinD,(aY,spinR)}	{spinD,(vZ0,spinR)}
C6	3.9408	spinD	(vZ0,spinR)	{spinD,(vZ0,z0)}	{spinD,vZ0}

**Table 14:** Top 12 ranked CEs of feature-sets across one-feature to three-feature settings on the Rosenberg Self-Esteem Test. CE of $Y(=Q_{1})$ is 1.2204.

One-Feature	CE	Two-Feature	CE	Three-Feature	CE
$Q_{2}$	0.8840	$Q_{2}\_Q_{6}$	0.8039	$Q_{2}\_Q_{4}\_Q_{6}$	0.7696
$Q_{6}$	0.9761	$Q_{2}\_Q_{3}$	0.8131	$Q_{2}\_Q_{3}\_Q_{6}$	0.7709
$Q_{7}$	1.0057	$Q_{2}\_Q_{7}$	0.8136	$Q_{2}\_Q_{6}\_Q_{10}$	0.7766
$Q_{4}$	1.0209	$Q_{2}\_Q_{10}$	0.8216	$Q_{2}\_Q_{3}\_Q_{7}$	0.7782
$Q_{3}$	1.0211	$Q_{2}\_Q_{4}$	0.8304	$Q_{2}\_Q_{3}\_Q_{4}$	0.7793
$Q_{10}$	1.0349	$Q_{2}\_Q_{5}$	0.8334	$Q_{2}\_Q_{4}\_Q_{7}$	0.7795
$Q_{5}$	1.0409	$Q_{2}\_Q_{9}$	0.8354	$Q_{2}\_Q_{5}\_Q_{6}$	0.7795
$Q_{9}$	1.0727	$Q_{2}\_Q_{8}$	0.8429	$Q_{2}\_Q_{7}\_Q_{10}$	0.7831
$V_{8}$	1.1141	$Q_{2}\_Age$	0.8806	$Q_{2}\_Q_{6}\_Q_{7}$	0.7831
Age	1.2002	$Q_{2}\_Gender$	0.8821	$Q_{2}\_Q_{6}\_Q_{9}$	0.7832
Gender	1.2133	$Q_{4}\_Q_{6}$	0.8908	$Q_{2}\_Q_{4}\_Q_{10}$	0.7849

**Table 15:** Top 12 ranked successive CE drops of feature-sets across one-feature to three-feature settings of the Rosenberg Self-Esteem Test.

One-Feature	SCE Drop	Two-Feature	SCE Drop	Three-Feature	SCE Drop
$Q_{2}$	0.3364	$Q_{3}\_Q_{4}$	0.1022	$Q_{3}\_Q_{4}\_Q_{7}$	0.0447
$Q_{6}$	0.2443	$Q_{4}\_Q_{10}$	0.0965	$Q_{4}\_Q_{7}\_Q_{10}$	0.0439
$Q_{7}$	0.2147	$Q_{4}\_Q_{7}$	0.0937	$Q_{4}\_Q_{5}\_Q_{10}$	0.0408
$Q_{4}$	0.1995	$Q_{4}\_Q_{5}$	0.0899	$Q_{3}\_Q_{4}\_Q_{6}$	0.0394
$Q_{3}$	0.1993	$Q_{4}\_Q_{6}$	0.0852	$Q_{3}\_Q_{4}\_Q_{10}$	0.0390
$Q_{10}$	0.1855	$Q_{2}\_Q_{6}$	0.0800	$Q_{4}\_Q_{5}\_Q_{7}$	0.0389
$Q_{5}$	0.1795	$Q_{2}\_Q_{3}$	0.0708	$Q_{4}\_Q_{6}\_Q_{10}$	0.0369
$Q_{9}$	0.1477	$Q_{4}\_Q_{9}$	0.0707	$Q_{2}\_Q_{4}\_Q_{10}$	0.0367
$V_{8}$	0.1063	$Q_{2}\_Q_{7}$	0.0704	$Q_{4}\_Q_{5}\_Q_{6}$	0.0361
Age	0.0202	$Q_{3}\_Q_{7}$	0.0703	$Q_{2}\_Q_{4}\_Q_{5}$	0.0354
Gender	0.0071	$Q_{5}\_Q_{10}$	0.0662	$Q_{2}\_Q_{3}\_Q_{7}$	0.0349

## Data Availability

The pitching data are available in PITCHf/x database belonging to Major League Baseball via http://gd2.mlb.com/components/game/mlb (accessed on 3 January 2022). Rosenberg Self-Esteem Scale data set from Kaggle can be found via https://www.kaggle.com/yamqwe/rosenberg-selfesteem-scale (accessed on 3 January 2022).
